# Supplementary figures and images for: Inequality indices to monitor geographic differences in incidence, mortality and fatality rates over time during the COVID-19 pandemic
Source: PLoS One. 2021 May 13;16(5):e0251366. doi: 10.1371/journal.pone.0251366 (PMC8118350; doi:10.1371/journal.pone.0251366)

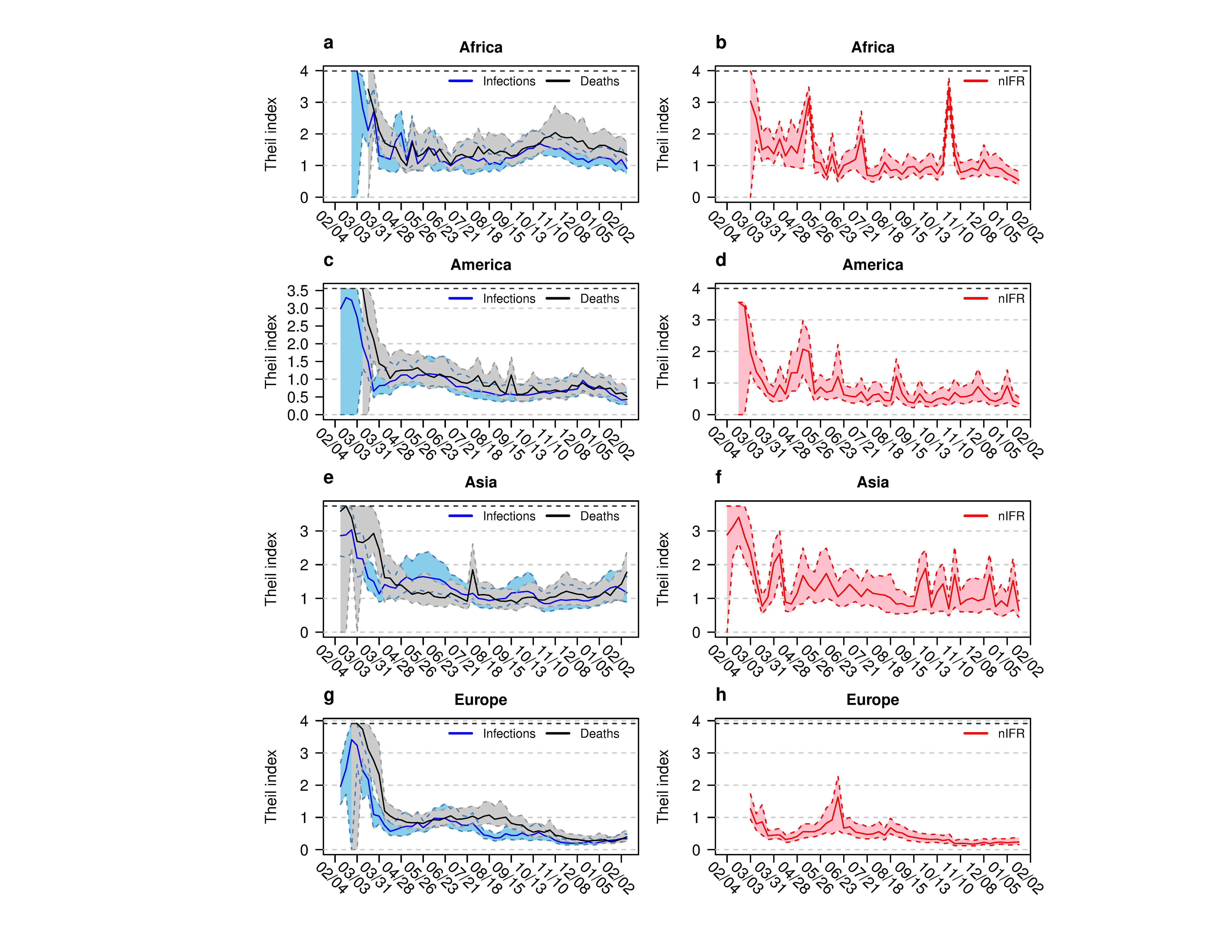

Supplement: S1 Fig — Time course of Theil index for infection and death rates (left column) as well as for naive infection fatality rate (nIFR, right column) is shown for Africa (a and b), America (c and d), Asia (e and f) and Europe (g and h). The coloured bands show the 95% confidence intervals. The horizontal axis shows the time between February 2020 and February 2021. The black dashed horizontal line shows the maximum possible value of the Theil index. (TIF) [file pone.0251366.s001.tif]

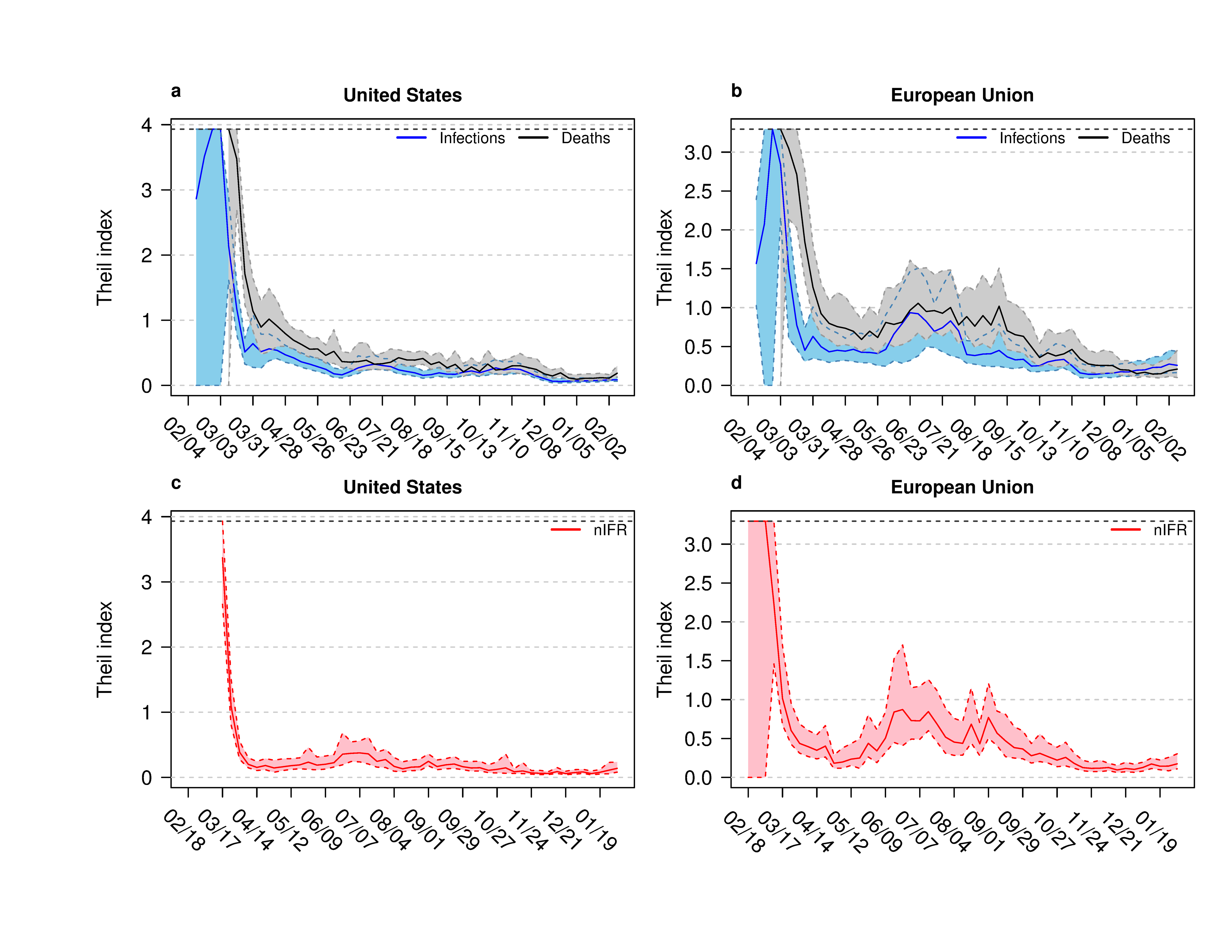

Supplement: S2 Fig — Time course of Theil index for infection and death rates (upper row) as well as for naive infection fatality rate (nIFR, lower row) is shown for the states of the United States (left column), and the member states of the European Union (right column). The coloured bands show the 95% confidence intervals. The horizontal axis shows the time between February 2020 and February 2021. The black dashed horizontal line shows the maximum possible value of the Theil index. (TIF) [file pone.0251366.s002.tif]
