# Supplementary material for: Inequality indices to monitor geographic differences in incidence, mortality and fatality rates over time during the COVID-19 pandemic
Source: PLoS One. 2021 May 13;16(5):e0251366. doi: 10.1371/journal.pone.0251366 (PMC8118350; doi:10.1371/journal.pone.0251366)
Supplement: S1 Table — Cumulative infection and death rates per 100000 inhabitants, cumulative number of infections and deaths, and the naive infection fatality rate in % are shown for the 27 current member states of the European Union as of February 2, 2021. (PDF) [file pone.0251366.s003.pdf]

S1 Table. Infection and death data for the European Union.

Country	Infection rate per 100000	Death rate per 100000	Infections	Deaths	nIFR (%)
Bulgaria	3151	131	220547	9178	4.2
Greece	1480	55	158716	5851	3.7
Italy	4259	148	2570608	89344	3.5
Hungary	3779	130	369288	12656	3.4
Belgium	6226	185	713271	21173	3.0
Germany	2698	71	2240017	58992	2.6
Poland	4003	99	1520215	37476	2.5
Romania	3774	95	732732	18513	2.5
France	4900	115	3283645	77383	2.4
Croatia	5714	124	232907	5071	2.2
Slovenia	8097	170	168486	3541	2.1
Spain	6076	127	2851869	59805	2.1
Sweden	5636	115	576606	11815	2.0
Austria	4705	89	416763	7847	1.9
Slovakia	4625	88	252094	4784	1.9
Latvia	3512	63	67427	1219	1.8
Portugal	7122	127	731861	13017	1.8
Czechia	9338	155	994514	16545	1.7
Ireland	4046	70	198424	3418	1.7
Lithuania	6565	102	183444	2847	1.6
Finland	830	12	45784	684	1.5
Malta	3681	55	18168	270	1.5
Netherlands	5780	82	998924	14218	1.4
Luxembourg	8295	95	50923	586	1.2
Denmark	3445	37	200041	2161	1.1
Estonia	3395	32	44978	424	0.9
Cyprus	3553	23	31124	202	0.6

Cumulative infection and death rates per 100.000 inhabitants, cumulative number of infections and deaths, and the naive infection fatality rate (nIFR) in % in the 27 member states of the European Union as of February 2, 2021.
