# Supplementary material for: Inequality indices to monitor geographic differences in incidence, mortality and fatality rates over time during the COVID-19 pandemic
Source: PLoS One. 2021 May 13;16(5):e0251366. doi: 10.1371/journal.pone.0251366 (PMC8118350; doi:10.1371/journal.pone.0251366)
Supplement: S2 Table — Cumulative infection and death rates per 100000 inhabitants, cumulative number of infections and deaths, and the naive infection fatality rate in % are shown for the U.S. states as of February 2, 2021. (PDF) [file pone.0251366.s004.pdf]

S2 **Table. Infection and death rates for the United States.**

State	Infection rate per 100000	Death rate per 100000	Infections	Deaths	nIFR (%)
New Jersey	7920	243	703496	21584	3.1
New York	7386	226	1436788	43982	3.1
Connecticut	7195	200	256522	7133	2.8
Massachusetts	7670	213	528662	14652	2.8
District of Columbia	5244	130	37008	916	2.5
Michigan	6150	156	614143	15609	2.5
Pennsylvania	6672	170	854197	21775	2.5
Louisiana	8695	193	404194	8953	2.2
Mississippi	9292	206	276531	6132	2.2
Maryland	5897	119	356541	7188	2.0
Illinois	8925	168	1130917	21336	1.9
New Mexico	8345	158	174982	3310	1.9
Rhode Island	10968	206	116191	2186	1.9
Alabama	9442	161	462938	7894	1.7
Arizona	10511	184	765083	13362	1.7
Arkansas	9875	164	298004	4939	1.7
West Virginia	6804	113	121935	2031	1.7
Georgia	8641	136	917440	14450	1.6
Hawaii	1852	29	26218	409	1.6
Indiana	9357	149	629903	10054	1.6
New Hampshire	4858	78	66058	1059	1.6
South Carolina	8699	142	447904	7318	1.6
South Dakota	12257	201	108431	1779	1.6
Florida	8090	125	1737640	26822	1.5
Iowa	10183	156	321274	4919	1.5
Maine	2973	46	39960	618	1.5
Missouri	7756	119	476020	7310	1.5
Nevada	9089	140	279957	4324	1.5
North Dakota	12833	190	97797	1447	1.5
Texas	8386	130	2431687	37619	1.5
Colorado	6912	98	398037	5650	1.4
Delaware	8082	114	78696	1108	1.4
Kansas	9582	130	279155	3797	1.4
Minnesota	8212	111	463132	6278	1.4
Oregon	3414	47	143978	1981	1.4
Tennessee	10709	145	731360	9900	1.4
Vermont	1955	28	12196	176	1.4
Washington	4133	57	314692	4316	1.4
Montana	8831	117	94384	1249	1.3
Ohio	7723	97	902736	11336	1.3
Virginia	5979	76	510380	6517	1.3
California	8493	106	3355781	41902	1.2
North Carolina	7287	90	764228	9409	1.2
Wyoming	9007	108	52128	624	1.2
Idaho	9158	97	163656	1741	1.1
Wisconsin	10206	111	594217	6480	1.1
Kentucky	8213	85	366929	3812	1.0
Nebraska	9896	100	191437	1929	1.0
Oklahoma	9911	91	392164	3602	0.9
Alaska	7463	38	54594	279	0.5
Utah	10868	53	348409	1685	0.5

Cumulative infection and death rates per 100000 inhabitants, cumulative number of infections and deaths, and the infection fatality rate (nIFR) in % in the U.S. states as of February 2, 2021.
