# Supplementary material for: Inequality indices to monitor geographic differences in incidence, mortality and fatality rates over time during the COVID-19 pandemic
Source: PLoS One. 2021 May 13;16(5):e0251366. doi: 10.1371/journal.pone.0251366 (PMC8118350; doi:10.1371/journal.pone.0251366)
Supplement: Attachment — Submitted filename: PONE-D-20-33079Rev2.pdf [file pone.0251366.s005.pdf]

PLOS ONE

In the face of the pandemic, are all equal? On the suitability of the Gini index to monitor time and geographic trends in incidence and death data during the SARS-CoV-2 pandemic

--Manuscript Draft--

Manuscript Number:	PONE-D-20-33079
Article Type:	Research Article
Full Title:	In the face of the pandemic, are all equal? On the suitability of the Gini index to monitor time and geographic trends in incidence and death data during the SARS-CoV-2 pandemic
Short Title:	Gini index and COVID-10 data monitoring
Corresponding Author:	Kirsi M. Manz, M.Sc.<br>Ludwig-Maximilians-Universitat Munchen<br>Muenchen, Bavaria GERMANY
Keywords:	SARS-CoV-2; COVID-19; Gini index; Gini coefficient; Inequality
Abstract:	Health inequalities across nations reinforce social and economic differences. The European Commission has outlined its commitment to reducing health inequalities. The SARS-CoV-2 pandemic is a disruptive event with global, national, and local effects on every aspect of a society's life. Therefore, it is of interest to quantify and display health (in)equality induced by COVID-19 over time and on different geographic scales (global, continental, national, regional).<br><br>We aimed to perform a study of (in)equality regarding COVID-19 related data on infections and deaths using the Gini index (GI). A specific focus of our analysis is the (in)equality of infection fatality rates. The Gini index was developed to explore inequality in wealth within or between nations. It is a relative measure which quantifies the ratio of the mean absolute difference between non-negative numerical observations and their mean: GI=0 describes a situation where all values are equal, GI=1 corresponds to one single positive observation among all non-negative observations. We use global, nation- and statewide data to evaluate the inequality at different geographical scales. We also reflect on the suitability of the Gini index to monitor equality aspects of the pandemic.<br><br>Data from the SARS-CoV-2 pandemic show a considerable amount of inequality in the global distribution of infections and deaths. Only few countries contribute largely to the total infection and death toll. Inequality may result from heterogeneous reporting systems, heterogeneous policy making to fight the pandemic, heterogeneous health systems, and demographic structures. Inequality decreases when going from continental to national or regional levels. Time trends at national level reflect local outbreaks. There is inequality in infection fatality rates, too. Because of equality of income or wealth within a society, low GIs (≤ 0.4) are related to positive connotations like fairness and justice. In the epidemiological setting, however, a GI=1 reflects the goal striven for: The epidemic is restricted to one region while the surroundings are not affected – the epidemic has failed to spread.<br><br>We used the Gini index to reflect inequality between incidence and death rates of nations or geographic regions. The interpretation of the differences between reported GIs is not straightforward. Furthermore, combined with the information presented by maps, the GI is a useful tool.
Order of Authors:	Kirsi M. Manz, M.Sc.
	Ulrich Mansmann
Additional Information:	
**Question**	**Response**

Powered by Editorial Manager® and ProduXion Manager® from Aries Systems Corporation

**Financial Disclosure**<br><br>Enter a financial disclosure statement that describes the sources of funding for the work included in this submission. Review the submission guidelines for detailed requirements. View published research articles from *PLOS ONE* for specific examples.<br><br>This statement is required for submission and **will appear in the published article** if the submission is accepted. Please make sure it is accurate.<br><br>**Unfunded studies**<br>Enter: *The author(s) received no specific funding for this work.*<br><br>**Funded studies**<br>Enter a statement with the following details:<br>• Initials of the authors who received each award<br>• Grant numbers awarded to each author<br>• The full name of each funder<br>• URL of each funder website<br>• Did the sponsors or funders play any role in the study design, data collection and analysis, decision to publish, or preparation of the manuscript?<br>• **NO** - Include this sentence at the end of your statement: *The funders had no role in study design, data collection and analysis, decision to publish, or preparation of the manuscript.*<br>• **YES** - Specify the role(s) played.<br><br>* typeset	The author(s) received no specific funding for this work.
**Competing Interests**<br><br>Use the instructions below to enter a competing interest statement for this submission. On behalf of all authors, disclose any competing interests that could be perceived to bias this work—acknowledging all financial support and any other relevant financial or non-financial competing interests.<br><br>This statement **will appear in the published article** if the submission is accepted. Please make sure it is	The authors have declared that no competing interests exist.

accurate. View published research articles from *PLOS ONE* for specific examples.<br><br>**NO authors have competing interests**<br><br>Enter: *The authors have declared that no competing interests exist.*<br><br>**Authors with competing interests**<br><br>Enter competing interest details beginning with this statement:<br><br>*I have read the journal's policy and the authors of this manuscript have the following competing interests: [insert competing interests here]*<br><br>* typeset	
**Ethics Statement**<br><br>Enter an ethics statement for this submission. This statement is required if the study involved:<br><br>• Human participants<br>• Human specimens or tissue<br>• Vertebrate animals or cephalopods<br>• Vertebrate embryos or tissues<br>• Field research<br><br>Write "N/A" if the submission does not require an ethics statement.<br><br>General guidance is provided below. Consult the submission guidelines for detailed instructions. **Make sure that all information entered here is included in the Methods section of the manuscript.**	N/A

**Format for specific study types**<br><br>**Human Subject Research (involving human participants and/or tissue)**<br>• Give the name of the institutional review board or ethics committee that approved the study<br>• Include the approval number and/or a statement indicating approval of this research<br>• Indicate the form of consent obtained (written/oral) or the reason that consent was not obtained (e.g. the data were analyzed anonymously)<br><br>**Animal Research (involving vertebrate animals, embryos or tissues)**<br>• Provide the name of the Institutional Animal Care and Use Committee (IACUC) or other relevant ethics board that reviewed the study protocol, and indicate whether they approved this research or granted a formal waiver of ethical approval<br>• Include an approval number if one was obtained<br>• If the study involved *non-human primates*, add *additional details* about animal welfare and steps taken to ameliorate suffering<br>• If anesthesia, euthanasia, or any kind of animal sacrifice is part of the study, include briefly which substances and/or methods were applied<br><br>**Field Research**<br><br>Include the following details if this study involves the collection of plant, animal, or other materials from a natural setting:<br>• Field permit number<br>• Name of the institution or relevant body that granted permission	
**Data Availability**<br><br>Authors are required to make all data underlying the findings described fully available, without restriction, and from the time of publication. PLOS allows rare exceptions to address legal and ethical concerns. See the PLOS Data Policy and FAQ for detailed information.	Yes - all data are fully available without restriction

Powered by Editorial Manager® and ProduXion Manager® from Aries Systems Corporation

A Data Availability Statement describing where the data can be found is required at submission. Your answers to this question constitute the Data Availability Statement and **will be published in the article**, if accepted.<br><br>**Important:** Stating 'data available on request from the author' is not sufficient. If your data are only available upon request, select 'No' for the first question and explain your exceptional situation in the text box.<br><br>Do the authors confirm that all data underlying the findings described in their manuscript are fully available without restriction?	
**Describe where the data may be found in full sentences. If you are copying our sample text, replace any instances of XXX with the appropriate details.**<br><br>• If the data are **held or will be held in a public repository**, include URLs, accession numbers or DOIs. If this information will only be available after acceptance, indicate this by ticking the box below. For example: *All XXX files are available from the XXX database (accession number(s) XXX, XXX.*).<br>• If the data are all contained **within the manuscript and/or Supporting Information files**, enter the following: *All relevant data are within the manuscript and its Supporting Information files.*<br>• If neither of these applies but you are able to provide **details of access elsewhere**, with or without limitations, please do so. For example:<br><br>*Data cannot be shared publicly because of [XXX]. Data are available from the XXX Institutional Data Access / Ethics Committee (contact via XXX) for researchers who meet the criteria for access to confidential data.*<br><br>*The data underlying the results presented in the study are available from (include the name of the third party*	All data can be found in the OSF data repository (https://osf.io/c3ren/).

and contact information or URL).<br>• This text is appropriate if the data are owned by a third party and authors do not have permission to share the data.<br><br>* typeset	
Additional data availability information:	

Manuscript Click here to access/download;Manuscript;Gini_PLOS_ONE_submission.pdf

In the face of the pandemic, are all equal? On the suitability of the Gini index to monitor time and geographic trends in incidence and death data during the SARS-CoV-2 pandemic

Kirsi Manz[1,*], Ulrich Mansmann[1],

1 Institute of Medical Information Processing, Biometry, and Epidemiology, Ludwig-Maximilians University Munich, Munich, Germany

* manz@ibe.med.uni-muenchen.de

Abstract

Health inequalities across nations reinforce social and economic differences. The European Commission has outlined its commitment to reducing health inequalities. The SARS-CoV-2 pandemic is a disruptive event with global, national, and local effects on every aspect of a society's life. Therefore, it is of interest to quantify and display health (in)equality induced by COVID-19 over time and on different geographic scales (global, continental, national, regional).

We aimed to perform a study of (in)equality regarding COVID-19 related data on infections and deaths using the Gini index (GI). A specific focus of our analysis is the (in)equality of infection fatality rates. The Gini index was developed to explore inequality in wealth within or between nations. It is a relative measure which quantifies the ratio of the mean absolute difference between non-negative numerical observations and their mean: GI=0 describes a situation where all values are equal, GI=1 corresponds to one single positive observation among all non-negative observations. We use global, nation- and statewide data to evaluate the inequality at different geographical scales. We also reflect on the suitability of the Gini index to monitor equality aspects of the pandemic.

Data from the SARS-CoV-2 pandemic show a considerable amount of inequality in the global distribution of infections and deaths. Only few countries contribute largely to the total infection and death toll. Inequality may result from heterogeneous reporting systems, heterogeneous policy making to fight the pandemic, heterogeneous health systems, and demographic structures. Inequality decreases when going from continental to national or regional levels. Time trends at national level reflect local outbreaks. There is inequality in infection fatality rates, too. Because of equality of income or wealth within a society, low GIs (≤ 0.4) are related to positive connotations like fairness and justice. In the epidemiological setting, however, a GI=1 reflects the goal striven for: The epidemic is restricted to one region while the surroundings are not affected – the epidemic has failed to spread.

We used the Gini index to reflect inequality between incidence and death rates of nations or geographic regions. The interpretation of the differences between reported GIs is not straightforward. Furthermore, combined with the information presented by maps, the GI is a useful tool.

Introduction

In his 1914 paper *Variability and Mutability* (Italian: *Variabilità e mutabilità*), the Italian statistician and sociologist Corrado Gini published a concept to represent the income or wealth inequality within a nation or any other group of people: The Gini Index (GI) [1]. If everybody receives the same income, the GI is equal to 0, in case that the total income of a nation is concentrated within one subject, the GI is 1. The Gini index only applies to non-negative values and cannot take negative income, negative wealth, or debts into account. It is sensitive to settings with a few very high and many low values.

Formally, the Gini index is the ratio of the mean absolute distance between all pairs of observed values and their arithmetic mean. This definition allows to deduce some heuristic insights in what this index is and provides. Especially, it is easy to see that the GI takes values between 0 and 1.

As mentioned above, a group of people where one person earns (owns) everything and all others have an income (property) of zero. Here, the GI is equal to one.

Economic development may increase the income (or property) of each person by the same amount. This leads to larger mean while the pairwise differences do not change. This results in a decrease of the GI, but does not change the variability (heterogeneity) of income within this group. Therefore, Gini's concept of inequality is different from the concept of variability: variability refers to heterogeneity, as represented by variance or standard deviation.

The arithmetic mean can be understood as the balance point of an abstract stick where the values are arranged as points of equal weight. The inner points can be moved towards their minimum and maximum without disturbing the balance. While keeping the arithmetic mean equal such a movement of the inner points increases (decreases) the mean absolute distance between all pairs of observed values and thus increases (decreases) the GI. This mind experiment explores the idea that the GI is related to inequality.

Inequality is also reflected by clusters. We characterize clusters by distances between the elements of a population: the distance between elements of different clusters is larger than the distance between the elements within a cluster. There is inequality between clusters and concentration within a cluster. The GI within a population with a large cluster of high and a small cluster of low income will be lower compared to the GI of a population with a large low and a small high income cluster. In both settings the mean absolute distance between all pairs may be equal. However, in the first setting the mean is close to the large upper cluster and in the second case close to the large lower cluster. Therefore, the GI is not symmetric and gives higher values for settings with a few high value observations. In economics the Gini index reflects *fairness* understood as equal opportunities for everybody. Comparable income is related to comparable access to resources.

In addition to the use of the Gini index in traditional economic settings it also plays a role in health-related topics where it is mainly applied to study *health equity.* Equity goals, such as equal treatment for equal need or equality of access, are central to health policy [2]. Specific applications of the GI regarding equity in health and health care are geographical inequality in mortality and life expectancy, and access to and distribution of health care resources [3–9]. The Gini index is also used to study the spread of infectious diseases. One article studies sexually transmitted diseases by investigating

their distribution according to level of sexual activity, another one is concerned with assessing inequality between malaria prevalences among populations [10, 11]. Another field of GI's application includes the analysis of the distribution of patient recruitment into clinical trials in critical care settings [12]. To the best of our knowledge, inequality in COVID-19 infection and death data has not been previously investigated in a quantitative manner using the GI.

This paper applies the Gini index to national and regional SARS-CoV-2 incidence and death measures. It explores the usefulness of Gini's inequality or concentration concept to interpret listings or maps of both measures. We argue that the index is a valuable tool for interpreting and comparing the inequality in disease load at different scales. However, it will be necessary to modify the GI's original interpretation.

A Gini index of zero in the economic setting is related to *fairness*: everyone owns the same. A Gini index of one describes the most unfair situation, where one persons owns everything. During an epidemic, however, a GI of one reflects the goal striven for: The epidemics is contained to one region while the ~~surroundings~~ are not affected – the epidemic has failed to spread. Furthermore, a low GI within a selection of regions expresses similarity or equality with respect to a common success or a common failure in disease control. Furthermore, it is of interest to study the GI over regions with a common health policy framework like the European Union (EU) or the United States of America (USA).

A property of the GI is that it is invariant with regard to multiplication: $\mathrm{GI}_{\mathrm{X}}=\mathrm{GI}_{\mathrm{Y}}$ for $X = a \cdot Y$ where X, Y are non-negative random variables and a is a positive number. The multiplicative invariance of the Gini index has a straightforward application to the infection fatality rate (IFR) of COVID-19: If the IFR is the same for all infected (over the range of populations considered) the Gini indices for incidence and death rates are the same: $\mathrm{GI}_{\mathrm{Infection}}=\mathrm{GI}_{\mathrm{Death}}$. Heterogeneity of demographic features such as age or preexisting comorbidities between regions can result in region-specific infection fatality rates. This will be reflected in the corresponding Gini indices. Given age and sex standardized rates, what is the meaning of an observation $\mathrm{GI}_{\mathrm{Infection}} < \mathrm{GI}_{\mathrm{Death}}$?

Altogether, we may expect the following from our project:

- A high GI may indicate a containment of the epidemic within regions under

consideration;

- A single high GI in a longitudinal observation over several regions reflects a local outbreak;
- A decrease of the GI over different regions indicates a growing similarity within these regions and may express a convergence within the regions either to the good (less disease load across the regions) or to the bad (having a common increase in cases);
- Absolute/relative numbers should accompany the interpretation of decreasing GIs over time in order to yield reliable interpretations, especially when the disease load is growing or decreasing.

Many publications and official reports provide maps and listings of regional SARS-CoV-2 incidence and/or death rates. Often there is no measure to quantify individual or global differences between these measures. To this end, *caterpillar plots* are sometimes used to represent estimates of the same parameter, such as death rate, and their variability. A caterpillar plot is a side-by-side bar plot of 95% confidence intervals for multiple estimates, where the estimates are ordered by their magnitude. These plots visualize variability and allow to compare measures and their uncertainty within a group of observational units. We will address this in our data. While the caterpillar plot represents the information hidden within listings in a better way, it lacks a global measure of inequality. Summarizing this discussion, we see the challenge and the potential gain when discussing the application of the Gini index for the interpretation of the dynamics of the COVID-19 pandemic. The ongoing pandemic offers a new interesting opportunity to apply this methodology known from the economy to a public health relevant area.

Materials and methods

The Data

For the analyses we use data from seven different sources. First, the global death and infection data were retrieved from the COVID-19 Data Repository by the Center for

Systems Science and Engineering at Johns Hopkins University (JHU CSSE) [13] containing data between January 29th and October 1st, 2020. The JHU CSSE collects and combines worldwide data related to COVID-19 infection and fatalities from many different sources.

Second, the population data for each country were extracted from the European Centre for Disease Control and Prevention (ECDC) COVID-19 database [14], where a population estimate for the year 2019 is provided. The data regarding the continents were also extracted from the ECDC data. These data were used for the global analysis (Fig 1, left column) and the analyses per continent (Fig 3).

Third, death and infection data for mainland of China were taken from the JHU CSSE data and included data on 31 Chinese provinces. Data for Hong Kong and Macao were excluded. Fourth, population estimates for the provinces were obtained from the Chinese National Bureau of Statistics of China for the year 2019 [15]. These data were used for Fig 1 (right column).

A subset of the JHU CSSE data was the basis for the analysis of the EU data, shown in Fig 2 (left column). The list of the current member states of the European Union was retrieved from the official website of the EU [16].

Fifth, the population data for each US state were obtained from the United States Census Bureau [17]. Puerto Rico was not included. A population estimate for the year 2019 was extracted. Combined with the data provided by the JHU CSSE Fig 2 (right column) was created.

Sixth, the data for Germany were downloaded from the Robert Koch Institute (RKI) COVID-19 dashboard [18]. We used the data provided on October 1st, 2020 and summarized the daily infection and death incidences separately for each German federal state (16 in total) and for each German administrative district (401 in total) for creating Fig 4. Seventh, the population data for each German district were obtained from the German Federal Statistical Office Destatis [19]. The most recent estimates for the year 2019 were used.

The regional data for the two German federal states Bavaria and North Rhine Westphalia (NRW) are a subset of the nationwide German data and contain infection and death incidences on a daily basis for 96 and 53 districts, respectively. Fig 5 was created using these data.

For all analyses we excluded any cruise ship data. Furthermore, for the Netherlands, United Kingdom and France we excluded data from all oversea territories (Netherlands: Aruba, Curacao, Sint Maarten, Bonaire, Sint Eustatius and Saba; UK: Bermuda, Cayman Islands, Gibraltar, Montserrat, Anguilla, British Virgin Islands, Turks and Caicos Islands and Falkland Islands (Malvinas); France: French Guiana, French Polynesia, Guadeloupe, Mayotte, New Caledonia, Reunion, Saint Barthelemy, St Martin, Martinique, Saint Pierre and Miquelon). Of note, due to the small number of affected countries in Oceania (N=4: Australia, Fiji, New Zealand and Papua New Quinea) the GI was not calculated. The corresponding confidence intervals would have been very wide reaching from 0 to 1 for many data points resulting in no meaningful estimation of the GI.

The data were analyzed using R Version 3.6.3 [20]. We used the DescTools package to calculate the GIs and the metafor package for the caterpillar plots. All data and R codes can be found in the OSF data repository (`https://osf.io/c3ren/`).

The disease measures

As described above we use data of population size combined with the data of the corresponding positive test results and fatalities related to COVID-19. For the interpretation of our results we have to take into account that the number of infected persons is different from the number of positively tested persons. Furthermore, the number of positively tested persons depends on the number of tests performed within a population. Therefore, the numbers used are biased estimates of the true incidences. Unfortunately, we do not have comprehensive data on testing strategies which would allow us to adjust the numbers used in our article towards better estimates. Therefore, the true COVID-19 prevalence, incidence, and mortality remain uncertain. In a recent preprint, Rahmandad et al. discuss implications of under-reporting across 86 nations on estimating COVID-19 disease measures [21]. Their estimated cumulative cases and deaths through 10 July 2020 are 1.47 up to 10.5 times official reports.

Formally, we work with the *test-positive fatality ratio* (*TPFR*), which is the ratio between documented COVID-19 related deaths and documented positively tested subjects. Different from this is the infection fatality rate (*IFR*), where the denominator

also includes all truly infected persons (with and without symptoms). Because of not tested infected persons without symptoms, it is plausible that IFR $\leq$ TPFR. In general, *TPFR* is set equal to *IFR*. Following the usual parlance we will also use the term *IFR*.

A recent meta-analysis pooling IFRs found an overall IFR estimate of 0.68% (95% confidence interval: 0.53% - 0.82%) [22]. However, the authors acknowledge that due to different demographic structures and perhaps underlying comorbidities, different populations might experience different IFRs from the disease.

To make the counts more comparable regarding the population size across the countries, we report incidence and death rates per 100000 persons. Due to lack of data we are not able to adjust these measures for sex and age. The time course data of Gini indices between relevant groups are studied in two ways: By a *periodic approach* presenting the numbers within a nation or region aggregated over non-overlapping short time periods of seven days and by a *cumulative approach* referring to the development of the numbers within a nation (or region) aggregated from the beginning up to time t. Corresponding bootstrap confidence intervals are also presented. Cumulative measures are presented in the Supplement, where we cumulate the data starting from January 28th, 2020 (global data) or April 16th, 2020 (German data) up to October 1st, 2020 (S1 Fig to S5 Fig). For better visualization, the GI of the fatalities is plotted one day later as the GI of infections.

The Gini index of inequality

The empirical *Gini* index can be calculated as

$$GI = \frac{\sum_{i=1}^{n} \sum_{j=1}^{n} |x_i - x_j|}{2n^2 \bar{x}}, \tag{1}$$

where x is an observed value (such as a specific incidence), n is the number of values observed and $\bar{x}$ is the mean value. Alternative formulae can be found in [23]. We use the bias-corrected bootstrap to calculate the confidence interval of a GI [24, 25].

The Gini index takes values between 0 and 1. The maximum value of 1 can ~~been~~ deduced from the inequality $|x - y| \leq |x| + |y|$. The Gini index is scale invariant. We combine this feature with the IFR: $D = p \cdot I$. Here D represents the incidence of death given the IFR (p) and the incidence of infected (I). Given this equality, it holds

$GI_{Infection} = GI_{Death}$.

On the other hand, the Gini index is not translation invariant. If a constant amount is added (subtracted) to (from) each value the Gini index decreases (increases). In terms of the pandemic, if all districts/countries succeed in lowering the death rate by the same constant amount, the corresponding Gini index would increase.

There are no established cut-offs to qualify specific GI values. It may be helpful to look at the quintile regions defined by 0.2, 0.4, 0.6, 0.8, and 1. This allows to have a neutral category in the middle and two ratings for inequality (strong: 0.6 to 0.8; high: 0.8 to 1) as well as equality (in terms of inequality low: 0.2 to 0.4; diminished; 0 to 0.2). The GI of the exponential distribution with rate 1 is about 0.5 and of the uniform distribution about 0.33. A gamma distribution with mean 20 and variance 40 has a GI of 0.18. The GI of a Pareto distribution with $\alpha = 0.7$ and threshold equal to 100 is close to 1.

We also combine the GI with maps and use it as a summary measure for the equality/inequality presented by the map.

Poisson regression for infection fatality rates

In order to evaluate if the IFRs between different regions are homogeneous we applied Poisson regression. The dependent variable is the absolute cumulative death count per observational unit (i.e. district, (federal) state or country) and the independent variable is the district itself. We used the absolute cumulative infection counts per region as an offset. The p value from the likelihood ratio test between the model and an offset-adjusted intercept only model was used to decide if the IFRs over the regions were homogeneous or not. The IFRs are shown in the Supporting Information (S1 Table – S4 Table).

Caterpillar Plots

Caterpillar plots are horizontal (vertical) plots that represent multiple ranked estimates together with their 95% confidence intervals. They provide a graphical impression of the variability within the estimates plotted and the precision of the estimation process. It may guide some hypotheses regarding which measure may be substantially different and

it offers a starting point for the posthoc analyses of differences within a group of comparable measures. For the COVID-19 data caterpillar plots are useful for presenting variability and differences within incidence and death rates.

Results

Overview of the global data

Up to September 29, 2020, globally around 33.64 million (33 643 204) SARS-CoV-2 infected and 1.00 million (1 007 740) deaths have been reported. The transmission of the virus has been documented in total of 186 countries and the reported number of infections per country varies between 10 (Western Sahara) and 7.19 million (United States). Deaths related to the virus have been documented in 173 countries and the number of deaths per affected country varies between 0 and 205 986. The US has worldwide the highest total number of infections and deaths (7 192 602 and 205 986) followed by India and Brazil with reported infections and deaths of 6.22 million and 142 921, and 4.78 millions and 97 497, respectively. The corresponding data can be found in the OSF data repository under `https://osf.io/c3ren/`.

In the following the GI time courses based on the *periodic approach* are presented. The results following the *cumulative approach* are presented as supplementary material (S1 Fig to S5 Fig).

Gini index for global and international data

Fig 1 provides the global view over 186 countries (left column) and the situation in the People's Republic of China (PRC, Mainland China, right column), where the pandemic started. Fig 1a shows the 7-day incidence rates per 100000 for each affected country. A prominent peak is seen for Qatar during May and June. Of all countries Israel shows the highest incidence rate with over 500 infected per 100000 inhabitants over one week (end of September, 2020). Regarding death rates (Fig 1b), San Marino shows the highest death rate. Another prominent peak can be seen for Ecuador. At the beginning of the epidemic, there are only a few infected in a specific region and most other regions have no infected or deaths. Therefore, at the beginning of the pandemic dynamics it is

natural that GI ~ 1 (Fig 1e). As the pandemic spreads into more regions, the Gini index starts to decrease from its starting value. Still, globally both indices remain at a high level.

Fig 1. Rates and periodic Gini index for global data and China. Infection rate, death rate and Gini indices (GIs) for global data (left column) and mainland China (right column). Panels a and b show the infection incidence rate per 100000 inhabitants over 7 days. Panels c and d show the death rate per 100000 inhabitants over 7 days. Panels e and f show the time course of GIs with their 95% confidence intervals describing incidence (black) and death data (blue) of the COVID-19 pandemic. The horizontal axis shows the calendar time between February 11th and September 29th, 2020. For better visualization the GIs of death estimates are shown one day apart from the GIs of infection estimates. The quintiles are shown as gray dashed lines.

The observations from mainland China show high inequality between the provinces: the vast majority of all infections and deaths were reported in the province of Hubei (Figs. 1b and d). This high inequality is reflected in the corresponding GI (Fig 1f). Of note, since no deaths were reported for China since end of June 2020 in any province, the GI for the death rates could not be calculated. The high peak introduced by the late correction of the number of reported deaths in Hubei does not largely affect the corresponding GI, since of all Chinese provinces the deaths mostly occurred in Hubei, anyway.

Fig 2 shows a comparison between the European Union (EU, left column) and the United States (US, right column). The upper rows show the incidence rates, the middle rows the death rates and the lower rows the corresponding GIs. Of the countries of the EU (Fig 2a) in the beginning of April a high peak in the incidence rate can be seen for Luxembourg. During April and May many EU countries show a peak in incidence rates, which then decline (probably due to the control efforts/lockdowns). Sweden has an incidence peak end of June, followed by a second peak in Luxembourg end of July. By the end of September Spain and Czechia have EU wide the highest incidence rates of around 200 per 100000 inhabitants. In Fig 2c the development of death rates with Belgium and Spain reporting the highest deaths rates during April and May can be seen. The corresponding GIs (Fig 2e) show less inequality than seen for the global data.

For the US (Fig 2b) New York (and New Jersey) reported the highest incidence rates in April. A second peak in many states is observed in July-August, where the highest incidence rates can be seen for Arizona and Florida. By the end of September North

Fig 2. Rates and periodic Gini index for EU and US. Infection rate, death rate and Gini indices (GIs) for the countries of the European Union (EU, left column) and the states of the United States (US, right column). Panels a and b show the infection incidence rate per 100000 inhabitants over 7 days. Panels c and d show the death rate per 100000 inhabitants over 7 days. Panels e and f show the time course of GIs with their 95% confidence intervals describing incidence (black) and death data (blue) of the COVID-19 pandemic. The horizontal axis shows the calendar time between February 11th and September 29th, 2020. For better visualization the GIs of death estimates are shown one day apart from the GIs of infection estimates. The quintiles are shown as gray dashed lines.

and South Dakota are the states with the highest incidence rates of around 500 per 100000 inhabitants. When it comes to death rates (Fig 2d), similarly to incidence rates, New York and New Jersey report the highest rates. The corresponding GIs (Fig 2f) are lower than the global GIs. When comparing the GIs for the EU and US (Fig 2e and Fig 2f) the US seems to have lower GIs (by one category) compared to the EU.

Additionally, the Gini index for different continents was evaluated. The *7-day* approach is shown in Fig 3. The GIs were calculated for N COVID-19 affected countries per continent (as of October 1st, 2020): Asia N=43, Europe N=49, America N=35, and Africa N=55. No Gini index is shown for Oceania due to the small number of affected countries (N=4). One should note that the continent of Europe contains more countries as the European Union (EU). For Asia (Fig 3a) the highest incidence rates

Fig 3. Incidence rates and periodic Gini index for continents. Infection incidence rates per 100000 over 7 days and Gini indices (GIs) for Asia (a, b), Europe (c, d), America (e, f) and Africa (g, h). The infection incidence rates are shown in the left column and the corresponding GIs in the right column. The GIs are shown together with their 95% confidence intervals describing incidence (black) and death data (blue) of the COVID-19 pandemic. The horizontal axis shows the calendar time between February 11th and September 29th, 2020. For better visualization the GIs of death estimates are shown one day apart from the GIs of infection estimates. The quintiles are shown as gray dashed lines.

were reported in June for Qatar and end of September for Israel. The corresponding GIs (Fig 3b) show high inequality between the different countries. The GIs for infections and deaths are comparable over the time. In Europe (Fig 3c) high incidence rate peaks during the spring can be seen for the Holy See (part of the Vatican City in Rome) and San Marino, both being regions with a low number of inhabitants. By the end of September Andorra (also with a low number of inhabitants) has the highest incidence rate of all European countries. The corresponding GIs (Fig 3d) show inequalities of

category 3 for infections and 4 for deaths. Interestingly, the inequality in deaths is higher than in infections since end of July 2020. In the Americas (Fig 3e) high incidence rates were reported for Chile (June), Panama (July) and Peru (August). By end of September Argentina is the country with the highest reported incidence rate of around 180 per 100000 inhabitants. The GIs for both infections and deaths remain comparable over time and of category 3 to 4 by the end of our observation period. The spread of the pandemic started later in Africa (Fig 3g). Here high incidence rates are seen for the small island countries of Sao Tome and Principe (in the Gulf of Guinea) and Cabo Verde (in the Atlantic Ocean). Of the larger countries South Africa has the highest incidence rates (up to 140 per 100000 inhabitants). In between lies Djibouti in the Horn of Africa with around 865 000 inhabitants. The corresponding GIs (Fig 3h) reflect high inequality of category 4 to 5. The GIs for both infections and deaths remain similar.

Gini index for Germany

The German data between April 16th and October 1st, 2020 were analysed. The first data point for the Gini index thus contains reported infections and deaths between April 16th and April 23rd, 2020. In Germany a total of 289 188 infections and 9 488 deaths have been reported up to October 1, 2020. The federal states Bavaria and North Rhine-Westphalia (NRW) are the two largest federal states which are together responsible for 48% of all reported infections and deaths (Bavaria: 24% of infections, 28% of deaths; NRW: 24% of infections,20% of deaths). The overall IFR is 3.3% for Germany, and 3.9% and 2.7% for Bavaria and NRW, respectively.

Fig 4 shows the incidence and death rates and the corresponding GIs for incidence and death rates over time for Germany with different regional foci: over the 16 federal states (left column) and over the 401 administrative districts (*Landkreise*, right column). Shown is also the risk threshold for the 7-day incidence of 50 per 100 000 population, which serves as a warning signal to further strengthen the local infection control measures. This threshold was introduced in May 2020. An alternative presentation of the statewide death data is presented in a caterpillar plot where observed death incidence estimates are ranked from lowest to highest and plotted together with their corresponding 95% confidence intervals (S6 Fig).

Fig 4. Rates and periodic Gini index for German federal states and districts. Infection incidence rate, death rate and Gini indices (GIs) for the German federal states (left column) and for the administrative districts (right column). Panels a and b show the 7-day infection incidence rate per 100000 inhabitants. Panels c and d show the 7-day death rate per 100000 inhabitants. Panels e and f show the time course of GIs with their 95% confidence intervals describing incidence (black) and death data (blue) of the COVID-19 pandemic. The horizontal axis shows the calendar time between April 23rd and October 1st, 2020. For better visualization the GIs of death estimates are shown one day apart from the GIs of infection estimates. The quintiles are shown as gray dashed lines.

The inequality between the federal states is lower than for the international data seen in Figs 1-3. When looking at the incidence rates at the district level (Fig 4b) two distinct local outbreaks can be seen: the orange-coloured peak end of June and the blue-coloured peak in August. The June peak refers to a large outbreak in a meat-processing factory in NRW with over 2000 infections. The August peak refers to two outbreaks in a district in Bavaria, where there was an outbreak in a farm among seasonal workers and a second one in a nearby canning factory. These two outbreaks can also be seen in the GI for infections, shown in Fig 4f. Also in Fig 4f distinct differences between $GI_{Infection}$ and GI_{Death} can be seen. Local outbreaks make single districts (*Landkreise*) for a specific period very distinct from the remaining districts, which increases the Gini index. Another smaller local outbreaks happened in nursing homes, factories, farms, and communal accommodation for refugees. An interesting observation is that the deaths are more unequally distributed than the infections. For infections, an increase in the GI can be observed until end of June; thereafter the inequality starts to decrease. The inequality within death rates increases and remains at a very high level of almost one (category 5). In the methods section we pointed to the fact that an equal IFR should result in equal GIs for both incidences.

A subset of the German data is shown in Fig 5 for the federal states Bavaria (left column) and NRW (right column). Bavaria has 96 and NRW 53 administrative districts. For better comparability the incidence and death rates are shown on the same scale. Both states show high epidemic activity compared to other German federal states.

The above mentioned outbreak can be seen Fig 5a, which is also reflected in the corresponding GI in Fig 5e. Also in Bavaria the deaths are very unequally distributed and the GI stays the most time in the highest category. The time course of the GI resembles the situation of German district level data shown in Fig 4f: Deaths are more

Fig 5. Rates and periodic Gini index for Bavaria and North Rhine-Westphalia. Infection incidence rate, death rate and Gini indices (GIs) for the German federal states Bavaria (left column) and North-Rhine Westphalia (right column). Panels a and b show the 7-day infection incidence rate per 100000 inhabitants. Panels c and d show the 7-day death incidence rate per 100000 inhabitants. Panels e and f show the time course of GIs with their 95% confidence intervals describing incidence (black) and death data (blue) of the COVID-19 pandemic. The horizontal axis shows the calendar time between April 23rd and October 1st, 2020. For better visualization the GIs of death estimates are shown one day apart from the GIs of infection estimates. The quintiles are shown as gray dashed lines.

unequally distributed than infections but the confidence intervals are wider (smaller number of administrative districts, N=96, compared to N=401). Of all German federal states Bavaria has the highest total number of infections and deaths per 100 000 inhabitants: The cumulative incidence rate per 100000 is 520 and death rate per 100000 is 20 as of October 1st, 2020 (for the rates also see S4 Table).

The right column of Fig 5 shows the data for the federal state of NRW. It is the largest federal state of Germany with nearly 18 million inhabitants. The large outbreak seen in the Germany wide data is clearly visible in Fig 5b and also reflected in the corresponding GI (Fig 5f). The inequality of incidence and death rates seems to resemble the situation of Bavaria: Both federal states seem to follow similar equality pattern reflected in similar time courses of $GI_{\mathrm{Infection}}$ and GI_{Death}.

Inhomogeneity of infection fatality rates

By means of Poisson regression and likelihood ratio test (LRT) we investigated if the IFRs are homogeneous over the geographical areas. As indicated by Table 1 inhomogeneous IFRs for all regions were found.

Gini index and disease maps

Gini indices can be combined with disease maps, whereas the GI serves as a summary measure for the inequality presented in the map. The maps correspond to the cumulative incidence data until October 1st, 2020. The global cumulative picture for infection and death incidences is shown in Fig 6. The corresponding GIs are 0.64 95% CI [0.60; 0.69] for infections and 0.71 95% CI [0.67; 0.75] for deaths. Both GIs represent strong inequality (category 4).

Table 1. Results of likelihood ratio test for homogeneity of infection fatality rates between different geographical regions.

Region	No. of observations	Type	p value (LRT)
World	186	countries	$<$**0.001**
Mainland China	31	provinces	$<$**0.001**
EU	27	countries	$<$**0.001**
US	51	states	$<$**0.001**
Asia	43	countries	$<$**0.001**
Europe	49	countries	$<$**0.001**
America	35	countries	$<$**0.001**
Africa	55	countries	$<$**0.001**
Germany	16	federal states	$<$**0.001**
Germany	401	districts	$<$**0.001**
Bavaria	96	districts	$<$**0.001**
North Rhine -Westphalia	53	districts	$<$**0.001**

The infection fatality rates were calculated using the cumulative infection and death data up October 1st, 2020. Significant p values are shown in bold. LRT = likelihood ratio test, EU = European Union, US = United States.

Fig 6. Global disease maps and the Gini index. Incidence rate map (blue-shaded upper map) and death rate map (red-shaded lower map). The corresponding GIs are 0.64 95% confidence interval (CI) [0.60; 0.69] for incidence rates and 0.71 95% CI [0.67; 0.75] for death rates. The maps were extracted from [26].

The second example presents national level data. Fig 7 represents cumulative infection and death incidences for Germany. The corresponding GIs are 0.32 95% CI [0.30; 0.35] for infections and 0.53 95% CI [0.50; 0.58] for deaths. Both GIs represent neutral and low inequality (category 3 and 2).

Fig 7. German disease maps and the Gini index. Incidence rate map (blue-shaded left map) and death rate map (red-shaded right map). The corresponding GIs are 0.32 95% confidence interval (CI) [0.30; 0.35] for incidence rates and 0.53 95% CI [0.50; 0.58] for death rates. The maps were extracted from [26].

Discussion

The aim of this paper is to explore the relevance of the Gini index to monitor SARS-CoV-2 infection and COVID-19 death incidences over time within different groups. We are interested in global, continental, and national views. The Gini index is a measure of *(in)equality* and sensitive to the relative distribution of low and high values within observations. It is high if only a few units are represented with large values and it decreases as more units occupy larger values. In economics equality in income or wealth

is a surrogate for equal opportunities and access to societal resources. Also during the SARS-CoV-2 pandemic disease measures like incidences of infections and death reflect differences within national systems and cooperative political strategies. For a given time interval, the Gini index can compare diverse geographical units (earth, continents, countries) and different regions or groups within a geographical unit (county). The comparison of distributions over time, the *periodic approach*, studies if inequalities are increasing or decreasing independently of absolute values. It is of interest if specific GI time courses reflect structural changes in strategies counteracting the pandemic.

In our first analysis we study the GI's time course for the entire world (Fig 1e) as well as mainland China (Fig 1f). Both panels show pronounced GI values towards inequality over time. While the situation in China is characterized by a containment of the epidemic within a few provinces (high values only in few provinces compared with many provinces without epidemic activities), the situation behind Fig 1e is different. Here the inequality is quantified between nations with distinct health care systems, incompatible administrative structures, and also disparate reporting processes. Countries with young populations (like India with a mean age of 28 years) may show a different death rate compared to countries with higher average age. Differences in demography will cause inequality regarding the disease measures of interest. While both GI time series (the global as well as the Chinese) show pronounced GI values their interpretation is different. Interestingly, the GIs that represent the inequality in death incidences are in both settings similar to the GIs corresponding to the infection incidences.

Fig 2 represents the corresponding GI time course for the countries of the European Union and the United States. GI values for death and infection incidences are higher in the EU compared to the US. Adapting to our rating scheme the EU GIs are one category above the US American GIs. As mentioned above this comparison does not allow to reflect on the reasons why the EU shows more inequality compared to the US. The GIs presented in both panels can not be compared regarding success or failure of handling the epidemic. In the EU as well as in the US, the single states operate independently of each other. The implementation of measures against COVID-19 in the US is in the responsibility of the state authorities and not of the federal government. It looks as if no federal state was especially successful in handling the epidemic. They look

equal with respect of lacking success. The US time course also presents two waves of spreading of the disease. The first occurred end of March 2020, the second wave started during June increasing the disease load. It is interesting to see that the US data shows lower death incidences during the second wave compared to the first wave. The clinical management of patients seems to be improved in general.

The comparison between continents (Fig 3) shows GI values above 0.5. Just based on optical similarity, Africa and Asia show comparable patterns as well as the Americas and Europe. What can be learned from this? The both GI time course profiles show high inequality and no specific time structure. Especially, the cut of inequality after the first phase of the pandemic (starting end of March) seems less pronounced in Africa and Asia compared to the Americas and Europe. This may be caused by a wider spread of the disease in the Americas and Europe.

The cumulative Asian data (S3 Fig) is the only situation where the inequality in infection incidences seems to be higher than the inequality in death incidences. This observation could be caused by the standardization of the data by using infection incidence and death rates, which were normalized to the respective population to gain a relative measure comparable across countries. Normalized rates for countries with low number of inhabitants can get large. For example the largest cumulative incidence rates in Asia (and also worldwide) as end of September were reported for Qatar and Bahrain (4433 and 4291 per 100 000, respectively). Both countries are small compared to other Asian countries (2.8 million and 1.6 million inhabitants) but contribute a substantial amount to the relative inequality, as measured by the Gini index in our study. It seems that the *relative* inequality between infection incidence rates in Asia is currently higher than in death rates but it remains to be seen if the situation will reverse again.

It is worth mentioning that all countries/regions contribute in an equal manner for the calculation of the GI. The only normalization we used was to adjust for the population to report rates per 100000 inhabitants, which were the quantities used to calculate the GI. We believe this approach is more suited than using absolute counts for the GI, since that way large countries would automatically contribute more than small countries. However, using the normalized rates we observe that often the highest rates were seen for small countries (Qatar, San Marino, Luxembourg, etc.). Perhaps assigning different weights to different sized countries and calculating a weighted Gini index could

provide an improvement to our approach [23].

We also present national data from Germany (Fig 4) and compare the data of two federal German states (Fig 5). As seen in Fig 4e, the German federal states show the lowest GIs in our data (GI ~ 0.3 which is comparable to the GI of the uniform distribution). During May 2020 Germany changed its policy by moving from a centrally guided approach to more regional (federal) policies. One may ~~hypothesise~~ that inequality increased during the federal disease control. However, after handing over the control to the authorities at district level, the GIs decrease. The federal GI measures for infections and deaths do not reflect this change in a pronounced way. Furthermore, inequality between death and infection incidences is more pronounced than at the federal level: $\text{GI}_{\text{Death}} > \text{GI}_{\text{Infection}}$. Across Germany, the absolute numbers of deaths decreased during the summer. There were many districts with zero reported deaths which forces the Gini index to increase.

Bavaria and NRW are the federal states with the largest populations within Germany. Both states are similar in the *cumulative* as well as the *7-day* approach. The latter shows specific local outbreaks like in a large meat processing factory (NRW) or at a farm and canning factory in Bavaria. We also observe that in NRW the GIs for infections recently further increase while the GIs for deaths remain at a very high level. During the summer vacation (in August 2020) Germany introduced general mass testing. This increased the number of tests used and the number of positively tested persons. Remembering that we do not adjust the infection incidence data for number of tests performed we stay with the same population size but increase the number of incident cases. As mentioned in the introduction this will decrease the GI. There may be the hypothesis that the decrease of district wide GIs starting August 2020 may also reflect the artificial increase in the total number of test positives caused by the change in the national testing strategies.

Generally, the data does not allow to adjust incidence and death rates for age and sex. It is well known that women and young persons have lower risk to die compared to men and people above 70 years [27]. Also the Gini index itself is not able to capture demographic variations among subgroups within the distribution, such as the distribution of deaths across age. By using a dichotomized age variable (< 60 years compared to ≥ 60 years old German population) we observed that the Gini indices for

both groups over German districts were not equal and thus the inequality can be probably partly explained by the age structure of the infected/deceased persons (S7 Fig). For sex no such pronounced difference was found (S8 Fig). As mentioned before, especially the death rate will be higher in older persons which results, as expected, in a lower GI compared to the GI of younger persons.

In summary, we observe that GIs decrease when the spatial scale of the analysis gets smaller. Inequality is more pronounced on the global scale compared to the situation within a nation or a continent. However, it is a known that the GI is downward-biased for small populations [28]. For diverse large population groups a higher GI is expected than for each of the sub-regions.

An interesting observation is that in many cases the inequality in the cumulative distribution of deaths is higher than the inequality in the distribution of cases. This was seen globally, for Europa (including the 27 countries of the EU) and for the US and also within Germany. A specific feature of the GI is its invariance with respect to a multiplication with a positive number. The number which compares incidences and deaths of an epidemic is the *infection fatality rate* IFR. A higher GI_{Death} compared to $GI_{Infection}$ may reflect heterogeneity of IFRs between the regions compared. The difference is more pronounced in the *cumulative* view. We found inhomogeneous IFRs over the regions considered.

Finally we combine the Gini index with a map. One useful feature of the GI is its *anonymity*. It does not disclose where the high and low values are. Combining the GI with a map removes this anonymity, but improves the interpretation of the map. Fig 6 presents worldwide infection (blue) and death (red) incidences. The choice of colour influences the way how one perceives and interprets the mapped information. The GI for infection incidences in blue looks less disperse as the death incidences presented in red. The corresponding GIs reflect the same impression. Overlapping confidence intervals may indicate no substantial difference in the corresponding inequalities. This is different in Fig 7 which presents the German data. While the inequality seems to be clearly different (non-overlapping CIs of the corresponding GI values), the graphical presentation does not allow this conclusion easily.

We also presented the caterpillar plot as an instrument to visualize differences and variability given in a listing or in a map.

Generally, the public discussion is directed towards the disease activity at a national or regional level. As is seems, only specialists are aware of the different aspects of the disease on a larger scale. For this reason we believe that tools are needed to represent the pandemic state of a nation/region within a broader framework. The economical and social consequences of the pandemic elsewhere will influence the own nation, too. It is of interest and also important to broaden the own horizon and get informed about the global development of the pandemic activity, not at least in order not to threaten the local progress in combating the consequences of the disease.

Conclusion

We found a considerable amount of inequality in the global distribution of SARS-CoV-2 related infections and deaths, as investigated using the Gini index. Only few countries contribute largely to the total incidence and death toll. Such inequality may result from heterogeneous reporting systems, different policies to fight the pandemic, heterogeneous health systems and demographic structures. When going from continental to national or regional levels the inequality in infections decreases. Distinct local outbreaks, however, increase the inequality, which can be reflected in the Gini index. There is inequality in infection fatality rates, too. In the epidemiological setting, a GI of one reflects the goal striven for: The epidemic is restricted to one region while the surroundings are not affected - the epidemic has failed to spread. The interpretation of the differences between reported GIs is not straightforward and has to be carefully framed. Altogether, combined with the information presented by maps/listings, the GI is a useful tool.

Supporting information

S1 Fig. Cumulative Gini index for global data and China. Time course of Gini indices (GIs) describing infection (black) and death data (blue) of the COVID-19 pandemic. The horizontal axis shows the calendar time between January 28th and September 15th, 2020. Shown are the global estimates (a) and estimates for mainland China (b) together with their 95% confidence intervals. For better visualization the GIs of death estimates are shown one day apart from the GIs of infection estimates. The

quintiles are shown as gray dashed lines.

S2 Fig. Cumulative Gini index for European Union and US. Time course of Gini indices (GIs) describing infection (black) and death data (blue) of the COVID-19 pandemic. The horizontal axis shows the calendar time between January 28th and September 15th, 2020. Shown are the estimates for the countries of the European Union (a) and for the states of the United States (US, b) together with their 95% confidence intervals. For better visualization the GIs of death estimates are shown one day apart from the GIs of infection estimates. The quintiles are shown as gray dashed lines.

S3 Fig. Cumulative Gini index for continents. Time course of Gini indices (GIs) describing infection (black) and death data (blue) of the COVID-19 pandemic. The horizontal axis shows the calendar time between January 28th and September 15th, 2020. Shown are the estimates for Asia (a), Europe (b), America (c) and Africa (d). The bars represent 95% confidence intervals. For better visualization the GIs of death estimates are shown one day apart from the GIs of infection estimates. The quintiles are shown as gray dashed lines.

S4 Fig. Cumulative Gini index for German federal states and districts. Time course of Gini indices (GIs) describing infection (black) and death data (blue) of the COVID-19 pandemic for Germany. The horizontal axis shows the calendar time between April 23th and October 1st, 2020. Shown are the estimates over the federal states (a) and over the administrative districts (b). The bars indicate 95% confidence intervals. For better visualization the GIs of death estimates are shown one day apart from the GIs of infection estimates. The quintiles are shown as gray dashed lines.

S5 Fig. Cumulative Gini index for Bavaria and North Rhine-Westphalia. Time course of Gini indices (GIs) describing infection (black) and death data (blue) of the COVID-19 pandemic for Germany. The horizontal axis shows the calendar time between April 23th and October 1st, 2020. Shown are the estimates for the federal state of Bavaria (a) and North Rhine-Westphalia (b). The bars indicate 95% confidence intervals. For better visualization the GIs of death estimates are shown one day apart from the GIs of infection estimates. The quintiles are shown as gray dashed lines.

S6 Fig. Caterpillar plot of death rates for German federal states. Shown are the cumulative death rates per 100000 inhabitants for the 16 German federal states together with their 95% confidence intervals as of October 1st, 2020. The corresponding Gini index is 0.33 95% confidence interval (CI) [0.25; 0.45].

S7 Fig. Sex dependency of the Gini index for Germany. Time course of Gini indices (GIs) describing infection (black) and death data (blue) of the COVID-19 pandemic for Germany. The horizontal axis shows the calendar time between April 16th and October 1st, 2020. Shown are the estimates for the federal states (a) and over the districts (b) for males (filled circles) and females (empty circles). The bars indicate 95% confidence intervals. For better visualization the GIs of death estimates are shown one day apart from the GIs of infection estimates. The quintiles are shown as gray dashed lines.

S8 Fig. Age dependency of the Gini index for Germany. Time course of Gini indices (GIs) describing infection (black) and death data (blue) of the COVID-19 pandemic for Germany. The horizontal axis shows the calendar time between April 16th and October 1st, 2020. Shown are the estimates for the federal states (a) and over the districts (b) for persons under 60 years or age (filled circles) and 60 years or older (empty circles). The bars indicate 95% confidence intervals. For better visualization the GIs of death estimates are shown one day apart from the GIs of infection estimates. The quintiles are shown as gray dashed lines.

S1 Appendix. Use of Gini index. The following PubMed search term can be used to gain more insight into the use of the Gini index:

Gini[tiab] AND equity AND financing AND (tax OR health care).

S1 Table. Infection and death data for China. Infection and death rates per 100000 inhabitants, the cumulative number of infections and deaths, and the infection fatality rate in % are shown for the 31 provinces of China as of October 1st, 2020.

S2 Table. Infection and death data for EU. Infection and death rates per 100000 inhabitants, the cumulative number of infections and deaths, and the infection

Table S1. Infection and death data in mainland China.

Province	Infection rate per 100000	Death rate per 100000	Infections	Deaths	IFR (%)
Hubei	115	8	68139	4512	6.6
Hainan	2	0	171	6	3.5
Henan	1	0	1281	22	1.7
Hebei	0	0	365	6	1.6
Guizhou	0	0	147	2	1.4
Heilongjiang	3	0	948	13	1.4
Jilin	1	0	157	2	1.3
Tianjin	2	0	240	3	1.3
Gansu	1	0	170	2	1.2
Beijing	4	0	936	9	1.0
Chongqing	2	0	585	6	1.0
Yunnan	0	0	209	2	1.0
Guangxi	1	0	259	2	0.8
Shandong	1	0	832	7	0.8
Liaoning	1	0	271	2	0.7
Shaanxi	1	0	410	3	0.7
Shanghai	4	0	1007	7	0.7
Anhui	2	0	991	6	0.6
Guangdong	2	0	1831	8	0.4
Hunan	1	0	1019	4	0.4
Inner Mongolia	1	0	266	1	0.4
Sichuan	1	0	696	3	0.4
Xinjiang	4	0	902	3	0.3
Fujian	1	0	411	1	0.2
Jiangxi	2	0	935	1	0.1
Zhejiang	2	0	1282	1	0.1
Jiangsu	1	0	666	0	0.0
Ningxia	1	0	75	0	0.0
Qinghai	0	0	18	0	0.0
Shanxi	1	0	204	0	0.0
Tibet	0	0	1	0	0.0

Infection and death rates per 100000 inhabitants, the cumulative number of infections and deaths, and the infection fatality rate (IFR) in % in the 31 Chinese provinces as of October 1st 2020. The corresponding Gini indices are 0.83 95% confidence interval (CI) [0.39; 0.90] for infection rates and 0.98 95% CI [0.96; 0.99] for death rates.

fatality rate in % are shown for the 27 current member states of the European Union as of October 1st, 2020.

S3 Table. Infection and death data for US. Infection and death rates per 100000 inhabitants, the cumulative number of infections and deaths, and the infection fatality rate in % are shown for the US states as of October 1st, 2020.

S4 Table. Infection and death data in Germany. Infection and death rates per

Table S2. Infection and death data for EU.

Country	Infection rate per 100000	Death rate per 100000	Infections	Deaths	IFR (%)
Italy	526	60	317409	35918	11.3
Belgium	1057	87	121059	10023	8.3
Sweden	915	58	93615	5893	6.3
France	921	48	616986	32034	5.2
Netherlands	748	37	129283	6470	5.0
Ireland	746	37	36597	1806	4.9
Spain	1659	68	778607	31973	4.1
Bulgaria	298	12	20833	825	4.0
Romania	668	25	129658	4862	3.7
Finland	183	6	10103	344	3.4
Germany	356	11	295539	9509	3.2
Hungary	279	8	27309	781	2.9
Poland	246	7	93481	2543	2.7
Portugal	743	19	76396	1977	2.6
Slovenia	282	7	5865	152	2.6
Denmark	497	11	28882	651	2.3
Greece	176	4	18886	393	2.1
Latvia	97	2	1868	37	2.0
Estonia	260	5	3450	65	1.9
Lithuania	171	3	4784	92	1.9
Austria	516	9	45686	802	1.8
Croatia	413	7	16827	284	1.7
Luxembourg	1400	20	8595	125	1.5
Cyprus	202	3	1772	22	1.2
Malta	627	7	3095	35	1.1
Czechia	697	6	74255	678	0.9
Slovakia	201	1	10938	48	0.4

Infection and death rates per 100000 inhabitants, the cumulative number of infections and deaths, and the infection fatality rate (IFR) in % in the 27 countries of the European Union as of October 1st 2020. The corresponding Gini indices are 0.38 95% confidence interval (CI) [0.32; 0.47] for infection rates and 0.57 95% CI [0.51; 0.67] for death rates.

100000 inhabitants, the cumulative number of infections and deaths, and the infection fatality rate in % are shown for the German federal states as of October 1st, 2020.

References

1. Gini C. Sulla misura della concentrazione e della variabilita dei caratteri. Atti del Reale Istituto Veneto di Scienze, Lettere ed Arti. 1914;73(2):1203-1248.

2. Le Grand, J. Equity, health, and health care. Soc Just Res. 1987;1:257–274. `https://doi.org/10.1007/BF01047663`

3. Yu H-Y, Chen J-J, Wang J-N, Chiu Y-L, Qiu H, Wang L-Y. Identification of the Differential Effect of City-Level on the Gini Coefficient of Health Service Delivery in Online Health Community. Int J Environ Res Public Health. 2019;16(13):2314. doi:10.3390/ijerph16132314.

4. Erdenee O, Paramita SA, Yamazaki C, Koyama H. Distribution of health care resources in Mongolia using the Gini coefficient Human Resources for Health. 2017;15:56. doi:10.1186/s12960-017-0232-1

5. Schwanke Khilji SU, Rudge JW, Drake T, Chavez I, Borin K, Touch S, et al. Distribution of selected healthcare resources for influenza pandemic response in Cambodia. Int J Equity Health. 2013;12:82. `http://www.equityhealthj.com/content/12/1/82`

6. Sun J, Luo H. Evaluation on equality and efficiency of health resources allocation and health services utilization in China. International Journal for Equity in Health. 2017;16:127. DOI:10.1186/s12939-017-0614-y

7. Zhang Y, Wang Q, Jiang T, Wang J. Equity and efficiency of primary health care resource allocation in mainland China. International Journal for Equity in Health. 2018;17:140. `https://doi.org/10.1186/s12939-018-0851-8`

8. Woldemichael A, Takian A, Akbari Sari A, Olyaeemanesh A. Availability and inequality in accessibility of health centre-based primary healthcare in Ethiopia. PLoS ONE. 2019;14(3):e0213896. `https://doi.org/10.1371/journal.pone.0213896`

9. Skaftun EK, Verguet S, Norheim OF, Johansson KA. Geographic health inequalities in Norway: a Gini analysis of cross-county differences in mortality from 1980 to 2014. Int J Equity Health. 2018;17:64. `https://doi.org/10.1186/s12939-018-0771-7`

10. Gsteiger S, Low N, Sonnenberg P, Mercer CH, Althaus CL. Gini coefficients for measuring the distribution of sexually transmitted infections among individuals with different levels of sexual activity. PeerJ 2020;8:e8434 DOI 10.7717/peerj.8434

11. Abeles J, Conway D. The Gini Coefficient as a useful measure of malaria inequality among populations https://www.medrxiv.org/content/early/2020/09/22/2020.09.19.20197939.

12. Ramanan M, Billot L, Rajbhandari D, Myburgh J, Finfer S, Bellomo R, et al. Does asymmetry in patient recruitment in large critical care trials follow the Pareto principle? Trials. 2020;21:378. https://doi.org/10.1186/s13063-020-04279-1

13. JHU CSSE COVID-19 Data Repository. https://github.com/CSSEGISandData/COVID-19. Accessed: July 1st, 2020

14. ECDC COVID-19 Data Repository. https://www.ecdc.europa.eu/en/geographical-distribution-2019-ncov-cases. Accessed: October 2nd, 2020

15. National Bureau of Statistics of China (NBS). http://data.stats.gov.cn/english/easyquery.htm?cn=E0103. Accessed: July 29th, 2020.

16. Official Website of the European Union. https://europa.eu/european-union/about-eu/countries_en. Accessed: July 20th, 2020.

17. United States Census Bureau. https://www.census.gov/data/tables/time-series/demo/popest/2010s-state-total.html. Accessed: July 24th, 2020.

18. Robert Koch Institut COVID-19 Dashboard. https://experience.arcgis.com/experience/478220a4c454480e823b17327b2bf1d4. Accessed: October 1st, 2020.

19. The German Federal Statistical Office. https://www.destatis.de/DE/Themen/Laender-Regionen/Regionales/Gemeindeverzeichnis/Administrativ/04-kreise.html. Accessed: July 22th, 2020.

20. R Core Team. R: A Language and Environment for Statistical Computing. R Foundation for Statistical Computing, Vienna, Austria. 2020. https://www.R-project.org/

21. Rahmandad H, Lim TY, Sterman, J. Estimating COVID-19 Under-Reporting Across 86 Nations: Implications for Projections and Control (August 3, 2020). Available at SSRN: https://ssrn.com/abstract=3635047

22. Meyerowitz-Katz G, Merone L. A systematic review and meta-analysis of published research data on COVID-19 infection-fatality rates. International Journal of Infectious Diseases. 2020. DOI: https://doi.org/10.1016/j.ijid.2020.09.1464

23. Cowell FA in Atkinson and Bourguignon (eds.). Measurement of Inequality. Handbook of Income Distribution. pp. 1-86, Elsevier, Amsterdam; 2000.

24. Dixon PM, Weiner J, Mitchell-Olds T, Woodley R. Bootstrapping the Gini coefficient of inequality. Ecology. 1987;68(5):1548-1551.

25. Efron B. The Jackknife, the Bootstrap, and Other Resampling Plans. CBMS-NSF Regional Conference Series in Applied Mathematics, Band 38. Capital City Press, Montpelier, Vermont, USA; 1982.

26. LMU StaBLab. https://corona.stat.uni-muenchen.de/maps/. Accessed: October 1st, 2020.

27. Williamson EJ, Walker AJ, Bhaskaran K, Bacon S, Bates C, Morton CE. Factors associated with COVID-19-related death using OpenSAFELY Nature. 2020. https://doi.org/10.1038/s41586-020-2521-4

28. Deltas G. The Small-sample Bias of the Gini Coefficient: Results and Implications for Empirical Research The Review of Economics and Statistics. 2003; 85(1):226-234.

Table S3. Infection and death rates for US.

State	Infection rate per 100000	Death rate per 100000	Infections	Deaths	IFR (%)
New Jersey	2318	182	205889	16140	7.8
Connecticut	1620	127	57742	4511	7.8
New York	2365	170	460031	33159	7.2
Massachusetts	1928	138	132870	9480	7.1
New Hampshire	612	32	8317	441	5.3
Michigan	1392	71	139012	7102	5.1
Pennsylvania	1289	64	165044	8144	4.9
Rhode Island	2352	105	24914	1117	4.5
District of Columbia	2172	89	15326	627	4.1
Louisiana	3583	119	166584	5519	3.3
Vermont	281	9	1755	58	3.3
Delaware	2135	65	20787	636	3.1
Maryland	2076	65	125510	3949	3.1
Ohio	1329	41	155314	4817	3.1
Mississippi	3323	100	98886	2979	3.0
Illinois	2351	71	297884	8940	3.0
Indiana	1800	54	121176	3645	3.0
New Mexico	1415	42	29661	882	3.0
Colorado	1236	36	71197	2054	2.9
Arizona	3012	78	219212	5674	2.6
Maine	404	10	5428	141	2.6
Washington	1157	28	88116	2132	2.4
South Carolina	2881	66	148323	3400	2.3
Georgia	3008	67	319359	7063	2.2
Virginia	1740	38	148536	3225	2.2
West Virginia	894	20	16026	359	2.2
Texas	2679	56	776736	16130	2.1
Minnesota	1777	37	100200	2102	2.1
Florida	3302	67	709144	14444	2.0
Nevada	2611	52	80410	1603	2.0
California	2081	40	822205	15973	1.9
North Carolina	2030	34	212898	3579	1.7
Kentucky	1561	27	69728	1191	1.7
Oregon	803	13	33862	560	1.7
Alabama	3176	52	155744	2548	1.6
Arkansas	2811	46	84821	1384	1.6
Missouri	2068	34	126906	2074	1.6
Iowa	2866	43	90428	1366	1.5
Tennessee	2891	37	197432	2501	1.3
Montana	1263	17	13500	181	1.3
North Dakota	2916	34	22218	256	1.2
Oklahoma	2233	26	88369	1035	1.2
Nebraska	2388	25	46185	493	1.1
Idaho	2382	26	42561	472	1.1
Wisconsin	2150	23	125161	1348	1.1
Kansas	2043	23	59514	672	1.1
Hawaii	884	10	12515	139	1.1
South Dakota	2615	27	23136	236	1.0
Wyoming	1051	9	6083	53	0.9
Alaska	1086	8	7948	57	0.7
Utah	2310	14	74050	459	0.6

Infection and death rates per 100000 inhabitants, the cumulative number of infections and deaths, and the infection fatality rate (IFR) in % in the US states as of October 1st, 2020. The corresponding Gini indices are 0.23 95% confidence interval (CI) [0.19; 0.29] for infection rates and 0.40 95% CI [0.34; 0.47] for death rates.

Table S4. Infection and death data for Germany.

Federal state	Infection rate per 100000	Death rate per 100000	Infections	Deaths	IFR (%)
Saarland	333	18	3297	177	5.4
Thuringia	190	9	4075	191	4.7
Brandenburg	170	7	4258	169	4.0
Bavaria	520	20	67988	2664	3.9
Baden-Württemberg	446	17	49418	1885	3.8
Hamburg	426	15	7837	270	3.4
Lower Saxony	253	9	20160	684	3.4
Schleswig-Holstein	165	6	4772	162	3.4
Saxony	177	6	7202	234	3.2
Hesse	302	9	18940	551	2.9
North Rhine-Westphalia	389	10	69814	1872	2.7
Saxony-Anhalt	119	3	2619	68	2.6
Bremen	357	9	2435	59	2.4
Rhineland-Palatinate	262	6	10698	254	2.4
Mecklenburg-Western Pomerania	73	1	1180	20	1.7
Berlin	398	6	14495	228	1.6

Infection and death rates per 100000 inhabitants, the cumulative number of infections and deaths, and the infection fatality rate (IFR) in % in the 16 German federal states as of 1st October, 2020. The corresponding Gini indices are 0.27 95% confidence interval (CI) [0.21; 0.40] for infection rates and 0.33 95% CI [0.25; 0.45] for death rates.

Figure 1

Click here to access/download;Figure;Fig1.eps

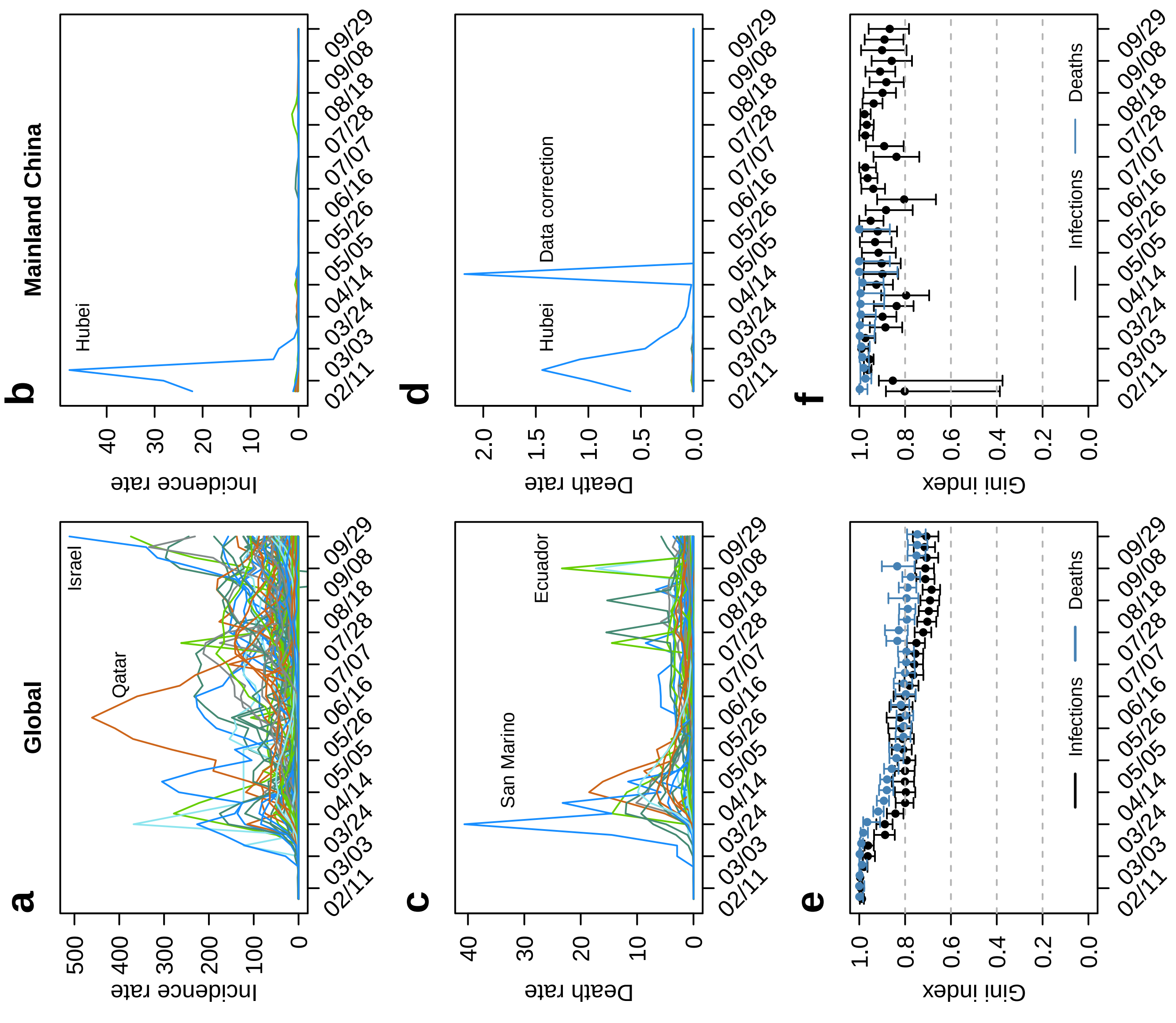

Click here to access/download;Figure;Fig2.eps

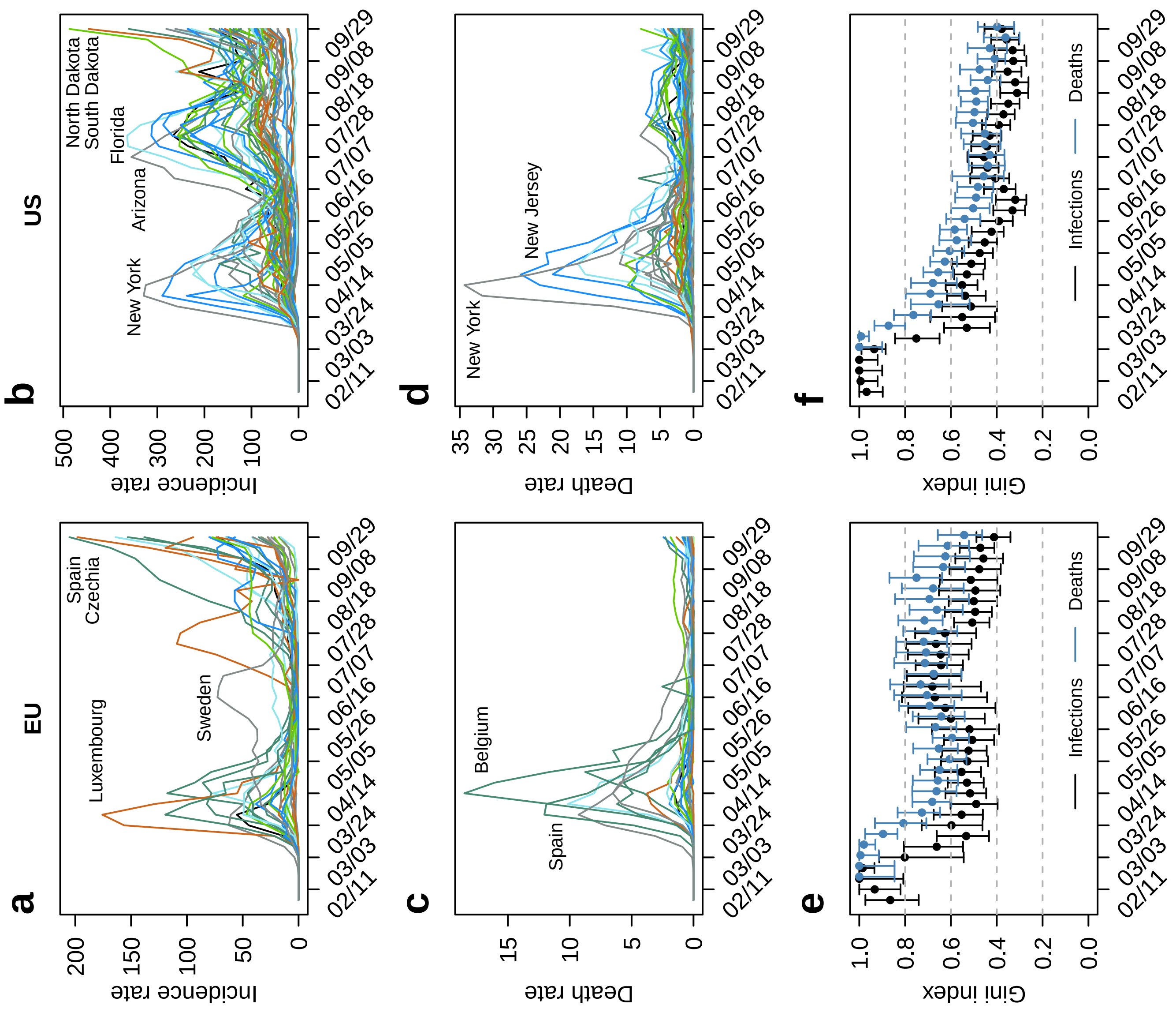

Figure 3 Click here to access/download;Figure;Fig3.eps

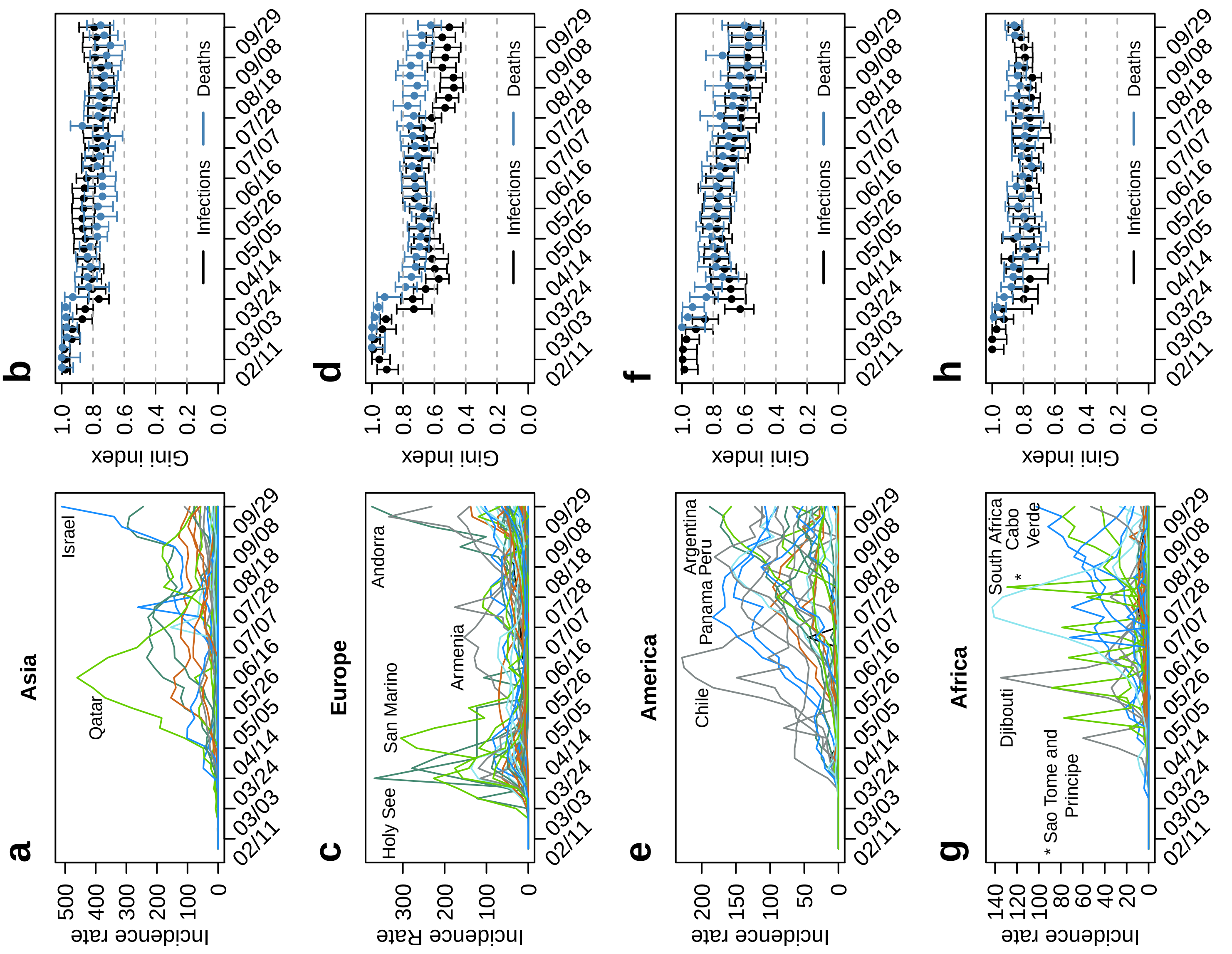

Figure 4

Click here to access/download;Figure;Fig4.eps

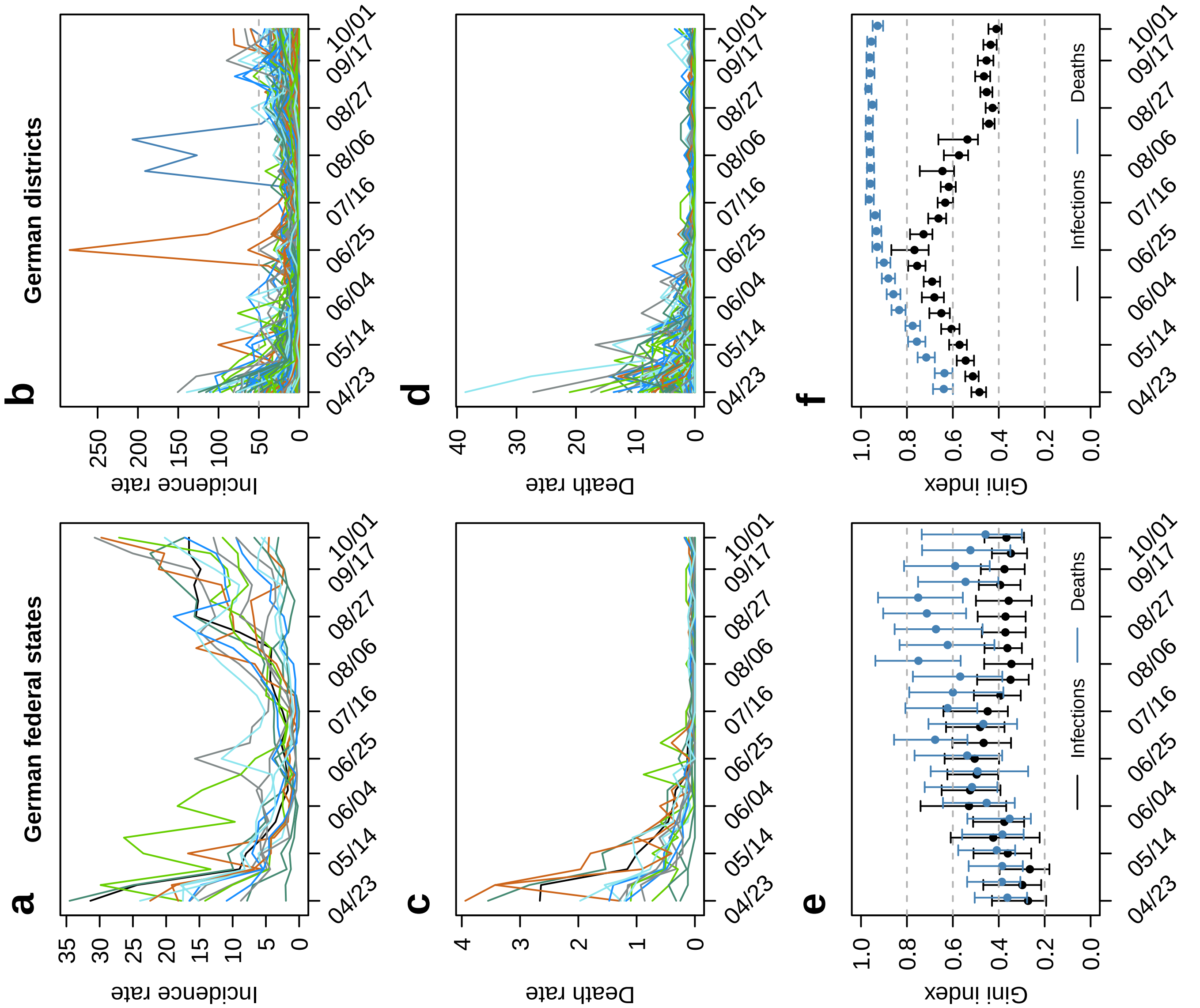

Click here to access/download;Figure;Fig5.eps

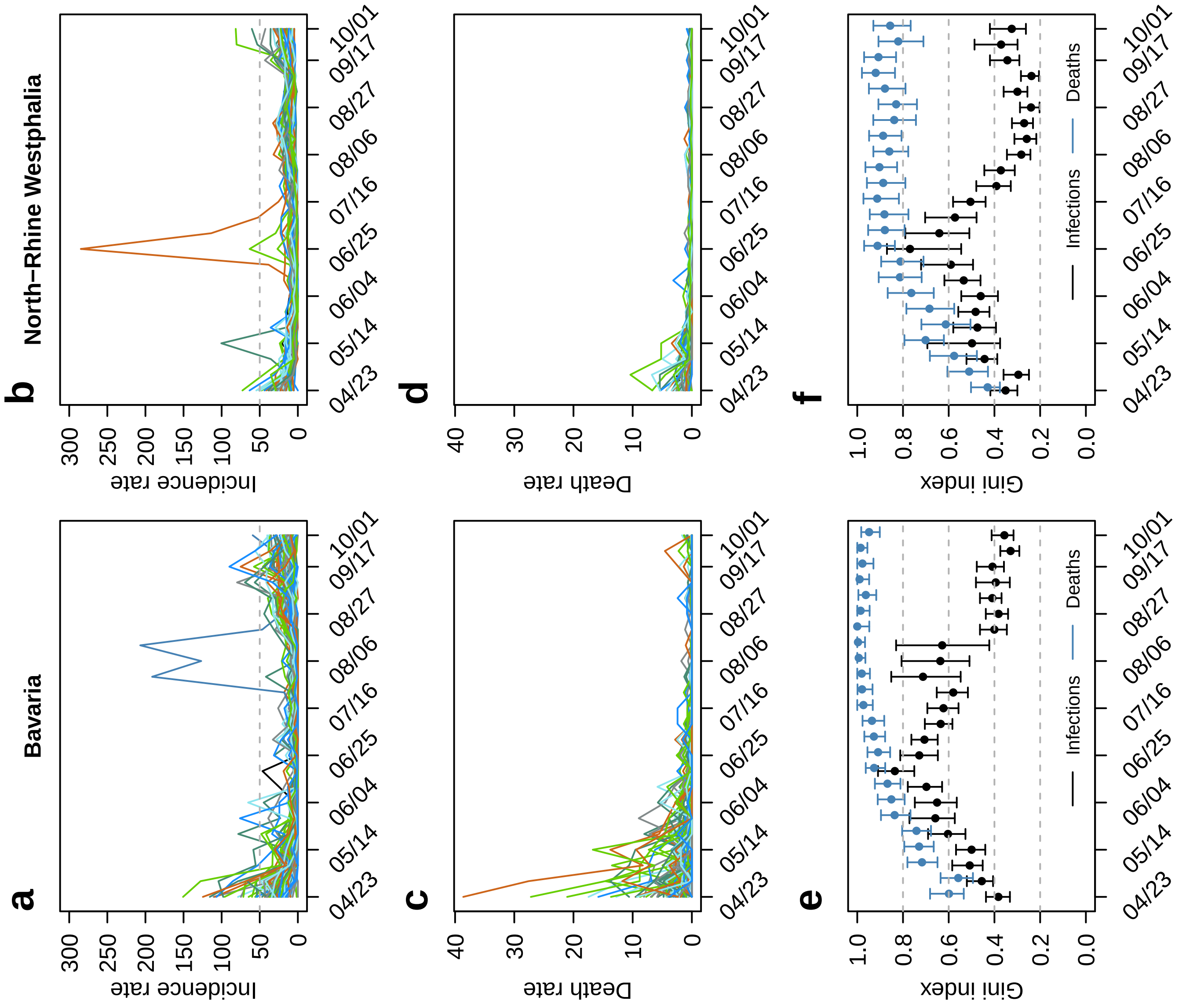

Figure 6

Click here to access/download;Figure;Fig6.eps

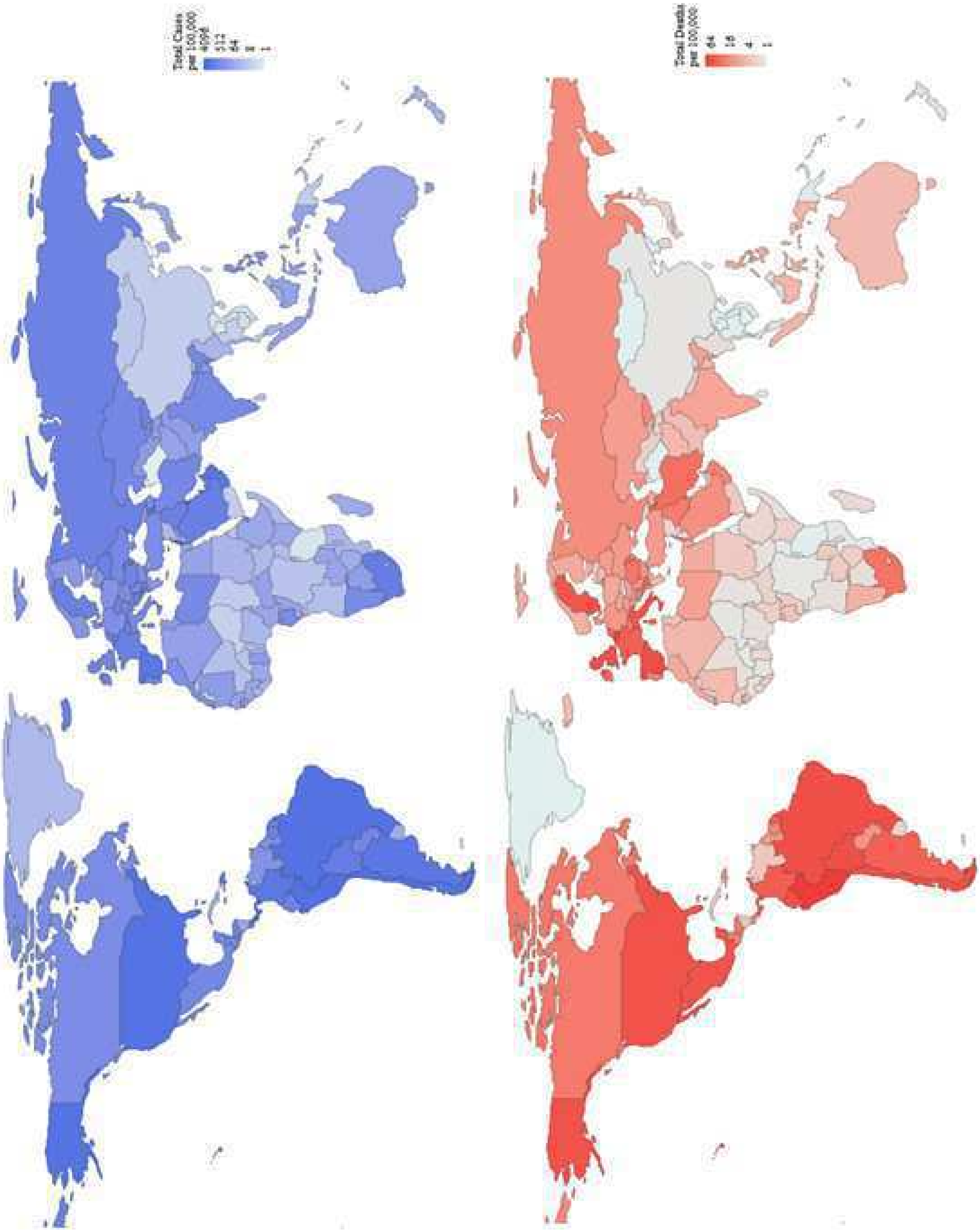

Figure 7

Click here to access/download;Figure;Fig7.eps

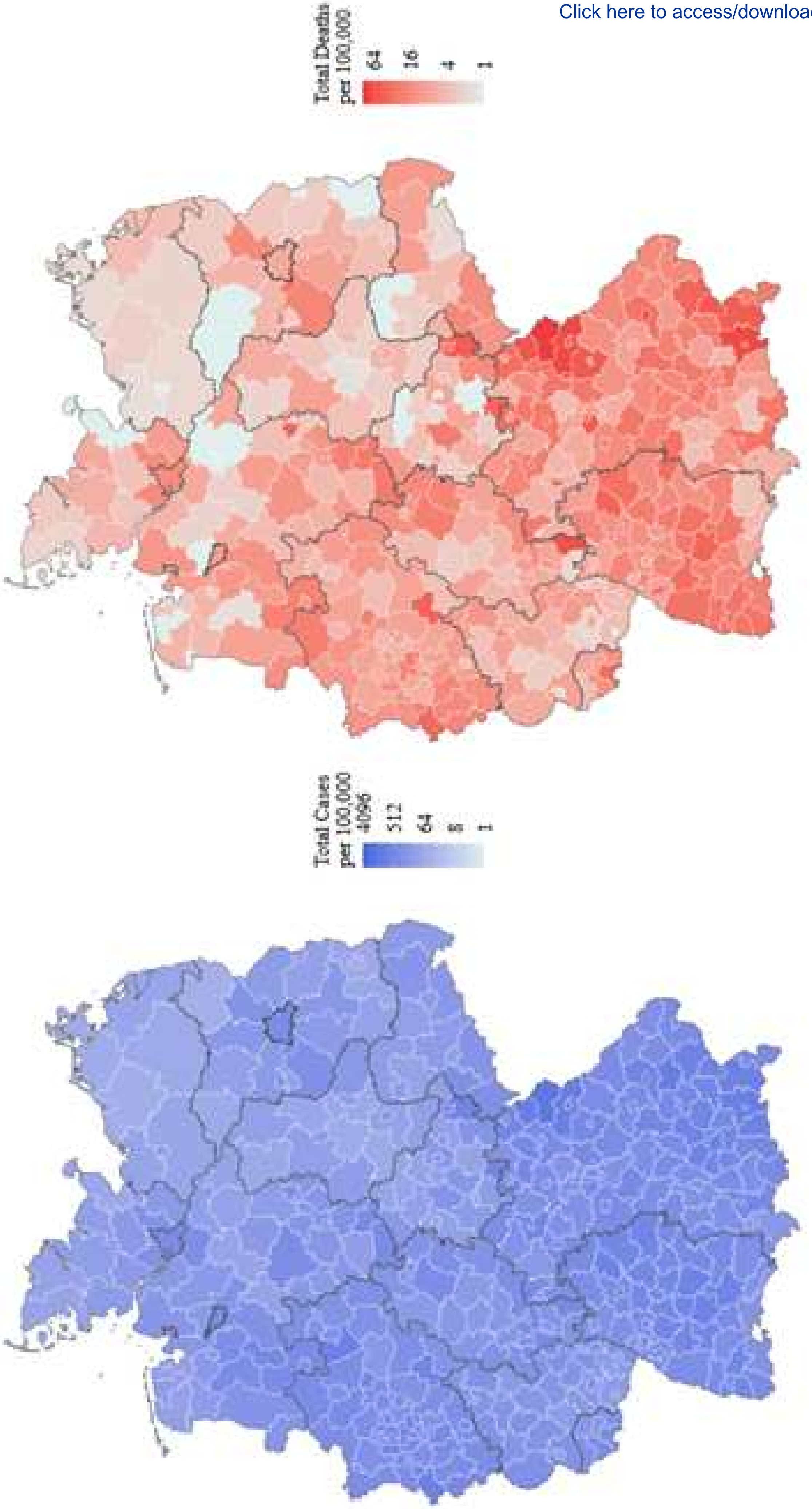

Click here to access/download
Supporting Information
S1_Fig.eps

Click here to access/download
Supporting Information
S2_Fig.eps

Click here to access/download
Supporting Information
S3_Fig.eps

Click here to access/download
Supporting Information
S4_Fig.eps

Click here to access/download
Supporting Information
S5_Fig.eps

Click here to access/download
Supporting Information
S6_Fig.eps

Click here to access/download
Supporting Information
S7_Fig.eps

Click here to access/download
Supporting Information
S8_Fig.eps
