# Supplementary material for: Inequality indices to monitor geographic differences in incidence, mortality and fatality rates over time during the COVID-19 pandemic
Source: PLoS One. 2021 May 13;16(5):e0251366. doi: 10.1371/journal.pone.0251366 (PMC8118350; doi:10.1371/journal.pone.0251366)
Supplement: Attachment — Submitted filename: Response_to_reviewers.docx [file pone.0251366.s006.docx]

**Response to reviewers' comments:**

***We would like to thank the reviewers for taking the time to read the manuscript and their valuable comments and suggestions on how to further improve our manuscript.***

***The comments given in the reviews motivated us to make a general reorganization of the paper. This concerns the following aspects:***

1. ***The abstract is structured (Background/Methods/Results/Conclusions)***
2. ***Essential shortening of the manuscript with focus on international aspects***
3. ***Broadening the spectrum of relevant inequality measures***
4. ***Extending the range of relevant endpoints***
5. ***Extending the time range to the first year of the pandemic.***

***Therefore, the revised manuscript is quite different from our first submission.***

***Here now the answers to the specific reviewers’ comments***

**Reviewer #1:** Dear authors,

Thank you for an interesting manuscript. It seems that you have used the best possible data for such a study, and the graphical presentation of your findings is really nice. However, I have some general comments, mainly addressing the choice of methodology and implications of your findings.

1. As I read your manuscript, my main concern is that I am not sure if you are applying an appropriate methodology and what the implications of your findings are. Differences in incidence and number of deaths between countries are not surprising findings, what does it add to analyze this using a Gini index? What are the moral implications? What are the policy implications? As you show in your manuscript, the interpretation of the Gini index is different for an infectious disease where high geographic inequality is desirable as this shows that the infection has not spread to all geographical areas. This makes inequality in COVID19 different from other health variables that previously have been studied using the Gini index. It seems a bit strange to analyze inequality in this manner (where the best outcome is high inequality), and I am not sure how this relates to fairness. As you discuss briefly in your manuscript, the difference between the observed inequality in incidence and deaths is explained by differences in the infection fatality rate (IFR). To me, this seems to be a highly relevant point to explore further (maybe even as the main topic of the paper), e.g. analyzing the inequality in IFR across geographic areas. Inequalities in IFR can be argued to be of moral significance (unfair), and such inequalities may also have policy implications as countries/regions should aim at the lowest possible IFR.

***Thanks for your comment. We used it as a starting point to revise the paper from the bottom. IFR is now a central topic.***

2. The Gini index is one of several inequality measures that could have been used for the analysis. Why do you find the Gini index to be the best summary measure for assessing inequality in incidence and mortality from COVID19? I think you should discuss and argue for why the Gini index is the best measure, as compared to other metrics that could have been used. Also, your introduction should contain some more information on findings from previous studies that have used the Gini index to assess geographic inequality.

***This is also a very valuable comment. Based on your input we took a broader look on alternative inequality measures and added the absolute Gini index and the Theil index. We present the results for all three indices and comment more critically on their interpretability. You are right in encouraging us to take a more critical view on the relative Gini index.***

***We also revised the part presenting the literature in health equity research in inequality measures.***

3. A couple of comments on how you have applied the Gini index to the data:

- Why did you choose the relative Gini index and not the absolute Gini index? Should we not be concerned with both the absolute levels and the inequality? The absolute and relative approach to inequalities both have their strengths and weaknesses, so I think you should include a brief discussion of why you have chosen a relative measure of inequality.

***Thank you for this valuable comment. We followed your advice and agreed to compare the relative and absolute Gini index. The relative Gini index seems to be a kind of conservative measure showing more equality if the absolute inequality increases.***

- Your analyses are done by country or region, without weighting for population size. I am not convinced that this is the most relevant approach, in my opinion we are concerned about inequalities between individuals living in different areas, not inequalities between the areas per se. If this is the case, I think the Gini index should be adjusted for population size.

***This is a valuable proposal. We decided to stay with equal weight per country approach because we are interested to assess the quality of the environment you life in and to compare them instead to introduce weights which would result in diminishing the influence of countries like the small European states in comparison to France or Germany, or in case of the US to weight the situation in states with large metropolitan areas in comparison to states with a more rural structure. Policy making is in general focused on countries and administrative/political units. This is the reason behind our approach.***

- The section about the Gini index’ scale invariance in the method part is confusing. The Gini index for infection will be equal to the Gini index of deaths if, and only if, the IFR is constant across all regions (which it is not).

***You are right and this issue is discussed in more detail now. We do present inequality measures for IFR rates. Actually we look at the naive IFR (nIFR) because we can not correct the reported infections regarding the missed infected persons.***

4. The manuscript is in general quite well written, but I think it could benefit from being a bit shorter and more concise, and additional language reviewing is still needed. Some parts need to be restructured, e.g. in the abstract the results should be presented before being discussed.

***We tried to shorten the manuscript essentially. We focus on the pandemic data of continents and perform a comparison between the U.S. and the EU. Regional data from Germany is removed and will be presented elsewhere.***

Good luck revising your manuscript!

**Reviewer #2:** The authors presented an interesting study to assess inequalities in COVID-19 transmission and deaths using as metric the Gini Index. The manuscript is generally well written and clear.

Major Issues:

Why the authors chose this index among others? Is there any specific characteristic of the index that makes it unique?

***The relative Gini index is the mostly used inequality index. This motivated our choice. However, as pointed out by the first reviewer, this classical index has problems and should be used with care. Therefore, we have extended our analyses and include both the absolute and relative Gini index as well as the Theil index.***

As stated by the authors, the different European states (or US states) adopt heterogenous policies to determine the effective number of deaths or infections. For this reason, is the Gini Index reliable if the infection and deaths are differently accounted?

***What we compare is quite complex. It is not only the true pandemic activity but also the impact of a health care system and its ways of documentation. The indices of inequality quantify differences but do not allow us to detect the sources of these differences. It is clear that specific countries in Africa may have an extensive pandemic situation which may not be documented since the corresponding infrastructure is missing.***

it seems that the analysis for Bavaria and districts show a different outcome with respect to the other results.

***This is correct and a well made observation. Unfortunately, this data is removed from the present manuscript In order not to overload the paper. We have now concentrated ourselves on comparing inequality between different continents and the U.S and the EU. But, if you look at the continental data you also see clear differences between Europe and Africa. They show quite different aspects of inequality within the specific region, but its interpretation has to be done with care. The comparison of inequality between Bavaria versus North-Rhine Westphalia may be much simpler since both federal states have comparable reporting systems and the observed inequality reflects internal differences regarding the epidemic course.***

An important factor that it is not mentioned it is the latitude dependency. As reported in literature, the different meteorological conditions impact on COVID-19 pandemic transmission. For this reason, instead of testing age and sex dependency, I would test latitude dependency.

***Thanks for this idea. Following you find a short analysis of this aspect. As a starting comment we would like to mention that the latitude-issue is confounded with specific continental views.***

***For example, subequatorial data is mixed from the Americas and Africa.***

***We assessed the latitude dependency by grouping the 190 countries together for 30° latitude bands. The latitude was extracted from the John-Hopkins-University COVID-19 data. Number of countries per latitude group:***

| Latitude (in °) | No. of countries |
| --- | --- |
| (60,90] | 5 |
| (30,60] | 66 |
| (0,30] | 81 |
| (-30,0] | 32 |
| (-60,-30] | 6 |
| (-90, -60] | 0 |

***We calculated the relative and absolute Gini index for infection incidences for the three most populated latitude bands. The figure shows the results.***

Specific comments can be found in the attached file.

***The comments provided in this additional file have been very helpful and have been fully considered in our revision.***

***Specific comments have focused on***

1. ***Weighted indexes: This issue is also raised by the first reviewer and answered above.***
2. ***Age adjustments: this is also commented in the limitations section of the paper - we simply had no data.***
